# An Ultra-Long-Acting Buprenorphine Prodrug Ion-Pair for Opioid Use Disorder

**DOI:** 10.21203/rs.3.rs-11194855/v1

**Published:** 2026-11-01

**Authors:** Benson Edagwa, Ashrafi Sultana, Nam Thai Hoang Le, Mohammad Ullah Nayan, Brady Sillman, Sunday Ogundepo, Chen Zhang, Brandon Hanson, Suyash Deodhar, Xiaoqing Du, Samuel Cohen, Brian Kearney, Alborz Yazdi, Howard Gendelman

**Affiliations:** University of Nebraska Medical Center; University Of Nebraska Medical Center; University of Nebraska Medical Center; University of Nebraska Medical Center; University of Nebraska Medical Center; University of Nebraska Medical Center; University of Nebraska Medical Center; University of Nebraska Medical Center; University of Nebraska Medical Center; University of Nebraska Medical Center; University of Nebraska Medical Center; Exavir Therapeutics Inc.; Exavir Therapeutics; University of Nebraska Medical Center

## Abstract

Opioid overdose is a significant global public health concern. While buprenorphine (BUP) is a safe and effective treatment for opioid use disorder (OUD), requirements for frequent dosing and patient tolerability to organic solvent based monthly injectables limit its use. These challenges were overcome by the development of a scalable ultra-long-acting (ULA) BUP (NM6BUP-Pamoate). The binding of a lipophilic BUP prodrug to a hydrophobic counterion generated an organic-solvent-free formulation. A single dose of NM6BUP-Pamoate administered to Sprague–Dawley rats at 45 mg/kg produced sustained effective plasma BUP levels for six months. Brain BUP concentrations exceeded the recorded plasma levels. Safety evaluations demonstrated the absence of off-target toxicities, with local and systemic tolerability. NM6BUP-Pamoate simplifies OUD treatment to optimize clinical outcomes.

## 1. INTRODUCTION

The recreational use of synthetic opioids is driving a global public health crisis. This crisis has led to both morbidity and mortality, with an immeasurable societal burden [1]. In the United States, opioid use disorder (OUD)-related adverse life events have reached record levels [2]. More than 17 million people are affected by OUD worldwide, resulting in more than 100,000 overdose fatalities and premature deaths annually [2]. Buprenorphine (BUP), a high-affinity partial μ-opioid receptor (MOR) agonist, is the cornerstone of OUD pharmacotherapy [3]. As a partial agonist with a ceiling effect on respiratory depression, BUP suppresses cravings and withdrawal symptoms. This leads to a lower intrinsic risk of fatal overdose compared to full μ-opioid receptor agonists such as methadone [4]. Clinically, BUP reduces illicit opioid use in patients with OUD. Importantly, observational studies have demonstrated significantly lower all-cause, drug-related, and suicide mortality rates. This ensues in BUP-treated individuals when compared to methadone, a full agonist [5, 6]. During active treatment, BUP is associated with a greater reduction in overdose risk than naltrexone [7]. This reflects sustained receptor occupancy and blockade of high-potency synthetic opioids. Despite these benefits, daily BUP dosing is limited by non-adherence to the regimen and medication diversion [8]. This significantly undermines real-world effectiveness. To address these treatment challenges, long-acting (LA) BUP formulations have been developed. These include the once-monthly biodegradable polymer-based formulation, Sublocade^®^, and the weekly or monthly liquid-crystal formulation, Brixadi^®^ [9, 10]. These therapies have been shown to improve adherence and treatment outcomes [11, 12]. However, the need for repeated clinical visits, limited access to treatment services, and adverse events limit their clinical impact [13, 14]. Injection-site reactions (ISRs) and tolerability have been linked to the use of non-physiological excipients or organic solvents [15], which limit patient acceptance. The six-month probuphine implant demonstrated efficacy comparable to that of other BUP formulations [16–18]. However, the need for invasive procedures, specialized training by healthcare providers, and high manufacturing costs resulted in product discontinuation, highlighting the need for minimally invasive ultra-LA (ULA) formulations [15].

To overcome the limitations of LA BUP injectables, we designed and screened a library of BUP free base ester prodrugs. We then created an ion pair with the lead candidate. Other reported lipophilic BUP ester prodrugs produce oily liquids that are incompatible with organic solvent-free scalable nanocrystalline extended-release formulations [19–21]. The lead conformationally restricted BUP free base prodrug, M6BUP, was successfully scaled up and produced free-flowing solid powders that were readily nanomilled into surfactant-stabilized aqueous suspensions (NM6BUP). Single intramuscular injections (IM) of NM6BUP administered at 45 and 90 mg/kg in Sprague Dawley (SD) rats produced dose-proportional therapeutic BUP concentrations for six months. At six months, brain levels of BUP remained high at 7.7 and 10.2 ng/g of brain tissue for 45 and 90 mg/kg doses, respectively, demonstrating efficient BUP CNS delivery. To further improve the formulation stability and drug concentration to lower injection volumes, M6BUP free base was ion-paired with pamoic acid to create M6BUP-Pamoate and the corresponding aqueous nanosuspension, NM6BUP-Pamoate. Notably, the BUP prodrug ion pair facilitated the scale-up and production of organic solvent-free formulations. This was achieved at drug concentrations of > 300 mg/ml compared to the maximum achievable concentration of 114 mg/mL for free-base prodrug suspensions. Similarly, a single 45 mg/kg subcutaneous (SC) dose of NM6BUP-Pamoate formulation in SD rats sustained therapeutic BUP concentrations for six months with no adverse events. The results paralleled those of prior clinically established aqueous-based LA injectable formulations for other chronic conditions, such as human immunodeficiency virus (HIV) infection and psychotic disorders [22, 23]. Similar implementation barriers, including reliable clinic access, available trained personnel, and societal stigma, affect real-world effectiveness [23]. For HIV, LA injectable antiretroviral therapies (ART) have demonstrated improved efficacy, enhanced patient satisfaction, and successful real-world scale-up across diverse healthcare systems [22]. Likewise, LA injectable antipsychotics improve adherence and clinical stability for mental illness, supporting the broader impact and acceptance of aqueous-based LA injectable suspensions [24]. Similar to other successful LA therapeutics, ULA NM6BUP-Pamoate demonstrates room-temperature stability for over six months. It can be scaled up for human dosing with low injection volume. Collectively, the potential of a dual prodrug ion pair approach led to a clinically feasible six-month aqueous-based BUP injectable formulation for opioid overdose.

## 2. RESULTS

### 2.1. M6BUP free base synthesis, scale up and off target toxicity profiles

Hydrophobic prodrugs extend drug exposure following IM or SC administration. This is achieved by forming a prolonged drug depot and slow prodrug dissolution [25]. The duration of drug exposure is linked to the prodrug chemical structure, particularly the hydrophobicity, linker chemistry, and solid-state properties. Conformationally restricted M6BUP was rationally designed to modulate the physicochemical properties of BUP to produce an aqueous-based formulation with prolonged therapeutic drug exposure compared to existing monthly injectables following extravascular administration. The design strategy leveraged the established safety profile of the selected arylated linker and was guided by our prior extensive investigations of lipophilic fatty acid–based prodrug [26–28]. M6BUP was synthesized using a two-step strategy, where in the first step, 4-aminobenzoic acid was acylated with stearoyl chloride to generate the arylated fatty-acid linker, 4-stearoylamidobenzoic acid (Fig. 1A). ^1^H NMR spectroscopy of the linker confirmed the characteristic resonances associated with the long-chain C18 fatty acid, including a terminal methyl signal (~ 0.85 ppm), a broad methylene signal (~ 1.20–1.40 ppm), and the appearance of a downfield amide N–H proton together with the aromatic proton peaks at ~ 7.6–10 ppm associated with para-substituted aminobenzoic acid (Supplementary Fig. 1). In the subsequent step, carbodiimide-mediated coupling of the purified linker to BUP yielded the target prodrug M6BUP, with a final chemical yield exceeding 70% (Fig. 1B). Structural confirmation of successful conjugation was achieved through comparative NMR analysis of the parent BUP, linker, and final prodrug. The ^1^H NMR spectrum of M6BUP retained the aromatic and aliphatic resonances of the BUP scaffold, and concurrently, new resonances corresponding to the linkage (~ 0.85–2.25 ppm) were observed, confirming the incorporation of the hydrophobic promoiety (Supplementary Fig. 2). Fourier-transform infrared (FT-IR) spectroscopy further identified prominent absorption bands at 2918 and 2854 cm^−1^ in M6BUP that were markedly attenuated in the parent BUP spectrum, corresponding to the asymmetric and symmetric methylene stretching vibrations from the long aliphatic chain (Fig. 1C). In addition, the appearance of a strong ester carbonyl stretch at 1751 cm^−1;^ confirmed the derivatization of BUP (Fig. 1C). Electrospray ionization (ESI) mass spectrometry further confirmed the molecular identity of M6BUP, yielding a dominant monoisotopic peak at m/z 853.6101, which matched the predicted molecular ion of the conjugate (Supplementary Fig. 3). Differential scanning calorimetry (DSC) revealed altered thermal transitions for M6BUP compared to BUP (Supplementary Fig. 4A). X-ray diffraction (XRD) analysis demonstrated a reduced long-range crystalline order for M6BUP compared with BUP. This was consistent with partial amorphization or disrupted crystal packing by the bulky hydrophobic substituents (Supplementary Fig. 4B). M6BUP stability from to 0-270°C during thermogravimetric analysis (TGA) further confirmed the anhydrous prodrug (Fig. 1D).

Establishing synthetic scalability is essential for ensuring the translational readiness of novel drug candidates. To confirm M6BUP translational scalability to support clinical testing and commercial production, M6BUP was produced on a 100-g BUP scale using the same chemistry and process parameters established at the bench scale. The isolated material exhibited ^1^H and ^13^C NMR spectra identical to those of the laboratory-derived product, confirming its structural consistency (Supplementary Fig. 5). The large-scale preparation afforded an overall yield of > 60% and demonstrated high chemical quality with 99.2% purity (Supplementary Fig. 6). Residual solvent analysis identified only trace amounts of heptane (< 0.05% w/w), and Karl Fischer moisture analysis confirmed 0.00% water content, indicating effective solvent removal and solid-state stability of the material. Collectively, these results demonstrate a robust and reproducible scale-up prodrug synthesis process that supports future translation.

Chemical modification of BUP to M6BUP substantially improved the physicochemical properties of the parent drug while preserving its functional activity towards target receptor occupancy. In a comprehensive cell-based functional panel screen (SAFETYscan E/IC_50_ ELECT–78) spanning 78 clinically relevant receptors, ion channels, transporters, kinases, and nuclear hormone receptors, M6BUP exhibited no measurable agonist, antagonist, inhibitory, or activation liabilities, indicating a favorable off-target safety profile and reduced susceptibility to clinically relevant adverse drug reactions (ADRs) (Supplementary Table 1). M6BUP efficacy towards MOR agonism remained comparable to that of BUP, with a 50% effective concentration (EC_50_) of 0.256 μM (Fig. 1E), suggesting efficient prodrug conversion. Importantly, M6BUP did not inhibit the human Ether-à-go-go-Related Gene (hERG) potassium channel at the maximum tested concentration of 10 μM, presenting no risk of clinical QT prolongation. As expected, aqueous and octanol solubility assessments confirmed decreased aqueous solubility for the prodrug (0.21 ± 0.01 μg/mL vs. 1.2 ± 0.1 μg/mL for M6BUP compared to BUP) (Fig. 1F). The octanol solubilities of M6BUP prodrug and BUP were 6039 ± 1470 μg/mL and 5625 ± 417 μg/mL, respectively (Fig. 1G). Additionally, M6BUP demonstrated sustained stability in both rat and human plasmas over 48 h (Fig. 1H). In rat and human plasma, 73.9 ± 2.5% and 66.7 ± 4.3% of M6BUP remained intact after 48 h, respectively. This pronounced shift toward lipophilicity and controlled hydrolysis is a defining characteristic of depot-forming prodrugs and is expected to promote slow prodrug dissolution, activation, and prolonged tissue retention following parenteral administration.

### 2.2. M6BUP free base aqueous nanosuspension production

To produce a well-tolerated aqueous-based parenteral formulation, surfactant-stabilized M6BUP prodrug nanosuspensions (NM6BUP) were developed using a scalable milling-compatible high-pressure homogenization process (Fig. 2). A key challenge in formulation process development is balancing drug loading with long-term formulation stability and syringeability. During the initial optimization process, the maximum achievable drug concentration following homogenization was limited to 40 mg/mL (4% w/v); beyond this, particle aggregation and loss of colloidal stability were observed. Therefore, this concentration represents the upper practical limit for producing a physically stable, injectable nanosuspension using this approach. The physical stability of NM6BUP was evaluated over an extended storage period of 145 d at room temperature (Fig. 2A). Throughout this period, the formulation maintained a mean particle size within a narrow range of approximately 208–261 nm, with corresponding polydispersity indices (PDIs) between 0.16 and 0.23, indicating a uniformly dispersed nano-suspension. Importantly, no evidence of flocculation, aggregation, or macroscopic phase separation was observed, and the formulation remained readily injectable throughout the study duration, indicating that NM6BUP possesses sufficient colloidal stability to support long-term storage and handling under conditions relevant for preclinical use. Although the initial nanosuspension exhibited excellent stability, the achievable drug concentration following homogenization alone imposed limitations on the injection volume for IM or SC administration, particularly in small animal models. To overcome this constraint, the nanosuspension was lyophilized and subsequently reconstituted in endotoxin-free water, enabling an increase in the effective formulation concentration while preserving particle integrity and injectability. This reconstitution strategy allowed the formulation to meet the allowable injection volume constraints for both IM and SC dosing without compromising the nanosuspension stability. Cellular safety, intracellular drug uptake, and retention of NM6BUP were evaluated in human monocyte-derived macrophages (MDMs), a clinically relevant cellular reservoir implicated in drug absorption from the injection site (Fig. 2C and D) [29]. Biocompatibility and cellular mitochondrial function assessment using the MTT assay demonstrated the safety profile of NM6BUP in MDMs at drug concentrations below 31.25 μM (Fig. 2B). For drug uptake studies, treatment of MDMs with 25 μM of M6BUP over a 24h study period revealed substantial intracellular delivery of the prodrug (465 ± 27.6 pmol/10^6^ cells), with measurable conversion to BUP (23.3 ± 2.8 pmol/10^6^ cells) at 24 h, indicating efficient cellular prodrug uptake and activation (Fig. 2C). To assess long-term intracellular drug retention, MDMs were exposed to 25 μM M6BUP for 8 h, followed by cell culture in drug-free media and drug quantitation at 1, 5, 10, and 20 days post-treatment (Fig. 2D). Intracellular M6BUP levels were sustained at 1067.4 ± 88.8, 418.4 ± 47.0, 591.0 ± 44.0, and 747.0 ± 61.0 pmol/10^6^ cells on days 1, 5, 10, and 20, respectively (Fig. 2D). Concurrently, intracellular BUP levels derived from prodrug activation were detected at 49.4 ± 3.8, 50.8 ± 1.4, 39.3 ± 1.3, and 17.6 ± 0.7 pmol/10^6^ cells at similar sampling intervals (Fig. 2D). These data indicate sustained intracellular retention of the prodrug with a gradual, timedependent release of active BUP. Transmission electron microscopy imaging further corroborated the intracellular fate of NM6BUP (Fig. 2E). At 8 h, 1 d, 10 d, and 20 d following treatment, electron-dense nanoscale structures were observed within intracellular compartments, consistent with the vesicular sequestration of the nanosuspension (Fig. 2E).

### 2.3. NM6BUP free base pharmacokinetics and biodistribution

The ULA PK profile of NM6BUP was evaluated following a single IM administration in SD rats at 45 (low dose) or 90 (high dose) mg/kg BUP-equivalent doses. Longitudinal observations were made for up to 182 days (Fig. 3). Across both dose levels, NM6BUP produced a prolonged plasma BUP profile, characterized by the absence of an initial burst release during the first seven days post-dosing (Fig. 3A). During this early absorption phase (days 1–7), the median plasma BUP concentrations remained low and stable, with median values of 3.05 (1.3–13.5) ng/mL and 3.9 (2.4–40.1) ng/mL for the low and high-dose groups, respectively. No statistically significant dose-proportional differences in plasma drug levels were observed on day 1 (two-tailed *t*-test with Welch correction, *p* = 0.3315), indicating controlled drug absorption. Following this initial lag phase, plasma BUP concentrations increased gradually between days 14 and 28, reaching maximal systemic exposure (C_max_) with a median T_max_ of 21 days for both dose groups, consistent with the slow absorption and release profile (Fig. 3H). The median plasma concentrations during the higher-absorption phase (days 14–28) were 11.65 (6.0–17.2) ng/mL and 16.75 (10.8–26.0) ng/mL for the low- and high-dose groups, respectively. Although statistically significant differences between the dose groups were observed at selected time points during this phase (days 28 and 56; *p* = 0.0336 and *p* = 0.015, respectively), and the cumulative exposure (AUC_0–∞_) was higher for the high-dose group (893.9 ± 41.3 vs. 530.0 ± 44.3 ng.day/mL; *p* = 0.0012), both groups exhibited a similar temporal pattern of extended systemic BUP exposure that persisted throughout the 182-day observation period (Fig. 3A, Supplementary Table 4).

After peak exposure, plasma BUP concentrations declined slowly and transitioned to a prolonged pseudo-plateau phase (Fig. 3A). From days 77 to 126, plasma concentrations remained within a narrow range, with median values of 1.1 (0.7–2.2) and 1.85 (1.4–2.6) ng/mL for the low- and high-dose groups, respectively. Steady BUP exposure persisted through the terminal phase of the study (days 133–182), during which the median plasma concentrations remained quantifiable at 0.56 (0.36–1.1) and 1.07 (0.6– 2.27) ng/mL for the low and high dose groups, respectively. Consequently, extended systemic exposure was reflected by prolonged elimination half-lives (T_½_), with median T_½_ values of 27.0 (14.5–44.0) and 42.1 (30.5–51.0) days for the low and high dose groups, respectively (Fig. 3H). The mean residence time (MRT_0–∞_) was similarly prolonged for both doses, with a significantly longer MRT observed at the higher dose (63.2 vs. 45.0 days; *p* = 0.0405). Throughout the study duration, intact prodrug concentrations in plasma remained at the limit of quantitation and became undetectable beyond day 143, indicating minimal systemic circulation of M6BUP relative to released BUP (Fig. 3B).

Consistent with the sustained systemic exposure, NM6BUP provided sustained tissue drug delivery. Brain BUP and M6BUP concentrations measured on day 56 for the low-dose group were 19.2 ± 5.1 ng/g, with low but detectable levels of M6BUP prodrug (6.8 ± 3.7 ng/g), indicating ongoing prodrug conversion and central nervous system exposure during the sustained phase (Fig. 3C). At day 182, brain BUP concentrations remained higher than the corresponding plasma levels, with concentrations of 7.7 ± 1.3 ng/g and 10.2 ± 1.1 ng/g for the low- and high-dose groups, respectively, indicating efficient drug delivery into the brain and CNS compartment (Fig. 3D). In parallel, analysis of the injection-site muscle at day 182 revealed persistent formulation material within phagocytic cells, supporting the presence of a prolonged drug depot (Fig. 3E–G). Throughout the entire observation period, the animals maintained stable body weights and serum chemistry within normal ranges, supporting the tolerability of the aqueous-based NM6BUP nanosuspension (Supplementary Fig. 7, Supplementary Table 2).

### 2.4. Synthesis, characterization and formulation of M6BUP-Pamoate prodrug ion pair

M6BUP was ion-paired with pamoic acid to generate a hydrophobic ion pair (HIP) prodrug M6BUP-Pamoate, thereby increasing the overall hydrophobicity without introducing any covalent modifications. This leveraged a reversible ionic association to enhance the physicochemical properties of the prodrug, enabling the production of substantially concentrated formulations that are not achievable with the M6BUP free base alone. As a result, the prodrug ion pair strategy produces ready-to-use (RTU) injectable formulations with reduced injection volumes at human-relevant scales. Reducing the injection volume is expected to improve patient comfort by minimizing injection-site discomfort and may support the broader clinical uptake of the formulation. The prodrug ion pair was synthesized through a single-step process by reacting solutions of the M6BUP prodrug and pamoic acid at a 2:1 molar ratio in dimethyl sulfoxide (DMSO), a polar aprotic solvent, followed by precipitation in water (Fig. 4A). The clinical safety of pamoic acid is well documented [30]. The structure of M6BUP-Pamoate was confirmed using ^1^H and ^13^C NMR spectroscopy and FT-IR analysis (Supplementary Fig. 8, Fig. 4B). Notably, hydrophobic ion pairing markedly enhanced the lipophilicity of the salt, with the M6BUP-Pamoate ion pair exhibiting an octanol solubility of 165.2 ± 3.0 mg/mL, representing a substantial increase relative to the M6BUP free base (Fig. 4C). This enhanced lipophilicity directly translated into improved drug loading capacity of the aqueous nanosuspensions, formulation stability, and syringeability. The scalability of the prodrug ion-pair-process was further demonstrated through multi-gram syntheses, demonstrating its translational potential. Structural analyses by FT-IR and ^1^H NMR verified the reproducible formation of the salt, maintaining the expected 1:0.5 stoichiometric pairing between M6BUP and pamoic acid (Supplement Figs. 9 and 10).

NM6BUP-Pamoate was formulated into an aqueous nanosuspension by high-pressure homogenization using the nonionic surfactants PEG-3350 and Tween-20. Pre-suspended NM6BUP-Pamoate in the surfactant solution was homogenized to produce a ready-to-use nanosuspension with a median particl size of 223 nm and a PDI of 0.2 (Fig. 4D). The resulting formulation achieved a drug loading of 300 mg/ml and remained physically stable over a 182-day observation period, with no evidence of flocculation, aggregation, or discoloration (Fig. 4D). Throughout this period, the particle size and PDI remained consistent, with median values of 223 (192–235) nm and PDI of 0.15 (0.10–0.20), and were readily syringeable using 28- and 25-gauge needles with consistent formulation appearance during storage (Fig. 4D). To further evaluate formulation scalability, a second aqueous based NM6BUP-Pamoate formulation with a higher final drug loading of 400 mg/ml was prepared and exhibited comparable particle size and stability characteristics over the observation period (Supplementary Fig. 11A). The cellular tolerability of the NM6BUP-Pamoate nanosuspension was assessed in MDMs using the MTT assay (Fig. 4E). As shown in Fig. 4E, NM6BUP-Pamoate demonstrated a safety profile comparable to that of the free-base NM6BUP formulation across the tested concentration range, with no significant reduction in cell viability. These findings indicate that hydrophobic ion pairing of the prodrug with pamoic acid does not adversely affect cellular mitochondrial activity.

### 2.5. NM6BUP-Pamoate ion pair formulation PK and biodistribution in SD rats

NM6BUP-Pamoate created from a HIP of M6BUP and pamoic acid, produced a ready-to-use concentrated formulation, allowing for reduced injection volumes compared to the free base formulation to improve injection site reactions (ISRs). This optimized formulation was evaluated in SD rats to assess the preservation of the ULA PK characteristics of the free base formulation over a six-month period following a single IM or SC administration at BUP-equivalent doses of 45 (IM- or SC-low) or 90 (IM-high) mg/kg (Fig. 5). Notably, across groups, NM6BUP-Pamoate produced prolonged plasma BUP levels that closely mirrored the temporal pattern observed with the NM6BUP free-base formulation while providing greater overall systemic exposure (Fig. 5A). In particular, the AUC_0–∞_ values were 815 ± 50.0 ng·day/mL (IM-low), 1271 ± 87.0 ng·day/mL (IM-high), and 661 ± 68.4 ng·day/mL (SC-low) (Fig. 5R), indicating efficient drug absorption relative to the NM6BUP free base under comparable conditions. NM6BUP-Pamoate effectively controlled the early initial burst release of BUP, where the median plasma drug concentrations on days 1 and 3 were 8.1 (4.2-22.01), 11.5 (7.3–17,1), and 5.0 (2.5–13.2) for IM-low, IM-high and SC-low dose, groups respectively. Compared to the NM6BUP free base formulation, a high absorption phase for NM6BUP-Pamoate was recorded from day 7 to day 14 post dosing. During this period, C_max_ was observed and ranged from 30.7 ± 5.7 ng/mL (IM-low), 43.8 ± 3.2 ng/mL (IM-high), and ± 2.5 ng/mL (SC-low), with corresponding T_max_ values of 14 (7–14) days for the IM-low group, 14 (14–14) days for the IM-high group, and 10.5 (7–14) days for the SC-low group (Fig. 5R). After day 14, plasma exposure transitioned into a steady decline rate, with drug levels quantifiable until day 182 (Fig. 5A). At days 28, 56, 91, 119, and 182, the mean plasma BUP concentrations following IM-low-dose administration were 7.9 ± 0.6, 5.2 ± 0.5, 1.8 ± 0.2, 0.9 ± 0.1, and 0.4 ± 0.0 ng/mL, respectively, whereas the corresponding concentrations following IM-high-dose administration were 16.9 ± 2.6, 4.9 ± 1.0, 2.3 ± 0.3, 1.1 ± 0.1, and 0.6 ± 0.1 ng/mL (Fig. 5A). Beyond day 28, no statistically significant differences were observed between the two IM dose levels, indicating broadly comparable terminal exposure profiles despite differences in the administered dose. Notably, plasma BUP concentrations following SC-low-dose administration closely mirrored those observed following IM dosing, with mean values of 7.6 ± 1.1, 2.4 ± 0.2, 1.5 ± 0.1, 1.2 ± 0.1, and 0.6 ± 0.1 ng/mL recorded on days 28, 56, 91, 119, and 182, respectively, supporting a similar LA exposure profile across alternate routes of drug administration (Fig. 5A). Beyond day 56, no statistically significant differences were observed between the mean plasma levels for the low-dose IM and SC groups.

Extended plasma exposure to NM6BUP-Pamoate translated into a prolonged terminal half-life (T_½_). The median T_½_ values were 45.7 (39.5–51.3), 41.6 (33.9–50.0), and 74.8 (70.6–79.0) days for the IM-low, IM-high, and SC-low dose groups, respectively (Fig. 5R), indicating the persistence of systemic BUP over several months. The prolonged MRT_0–∞_ for all groups was 42.11 days (40.8–45.3), 41.39 days (37.3–47.0), and 73.6 days (64.1–82.7) for the IM-low, IM-high, and SC-low dose groups, respectively (Fig. 5R). Notably, the NM6BUP-Pamoate nanoformulation achieved sustained brain delivery of BUP, with brain concentrations exceeding plasma levels at matched time points (Fig. 5B). At day 182, the mean brain BUP concentrations were 4.9 ± 0.36, 6.55 ± 0.97, and 5.18 ± 0.68 ng/g for the IM-low, IM-high, and SC-low dose groups, respectively. Animal weights and serum chemistry parameters indicated no systemic side effects of the NM6BUP-Pamoate formulation (Supplementary Fig. 12 and Supplementary Table 3).

To further characterize early brain distribution, regional brain BUP concentrations were evaluated following IM or SC administration at a higher dose. Substantial BUP levels were detected across multiple brain regions as early as day 1 and remained consistently measurable throughout the early observation period (Fig. 5C–E). In the cortex, the mean BUP concentrations following IM administration were 23.8 ± 1.6 ng/g on day 1, 29.9 ± 0.9 ng/g on day 3, and 36.6 ± 4.1 ng/g on day 7. For SC administration, the BUP concentrations in the cortex at similar time points were 14.5 ± 1.6, 22.5 ± 1.0, and 18.5 ± 2.3 ng/g, respectively (Fig. 5C). In the cerebellum, IM dosing produced mean BUP concentrations of 14.2 ± 2.2 ng/g (day 1), 19.4 ± 1.8 ng/g (day 3), and 22.1 ± 3.2 ng/g (day 7), whereas SC administration resulted in 7.7 ± 1.2, 13.2 ± 3.0, and 8.7 ± 2.0 ng/g at the corresponding time points (Fig. 5D). Similarly, in the midbrain, IM administration produced mean BUP concentrations of 36.3 ± 1.5 ng/g on day 1, 43.5 ± 0.5 ng/g on day 3, and 42.0 ± 8.6 ng/g on day 7, compared with 22.4 ± 1.4, 33.8 ± 3.7, and 27.7 ± 1.6 ng/g following SC administration (Fig. 5E). To determine the potential for brain toxicity associated with BUP exposure from NM6BUP-Pamoate administration, histological evaluation of brain tissues was performed by an experienced board-certified pathologist and toxicologist after hematoxylin and eosin (H&E) staining. Sections of the cortex and midbrain collected on days one and 14 following treatment were compared with vehicle-treated and no-injection controls (Fig. 5F–Q). Tissue architecture was preserved in both brain subregions, with no apparent treatment-histopathological alterations, supporting brain tissue integrity following formulation treatments.

## 3. DISCUSSION

Opioids are critical for managing moderate-to-severe acute, cancer-related, and end-of-life pain. However, long-term or excessive use leads to lasting physical, neurological, and mental health complications, including tolerance, dependence, and overdose [31, 32]. However, their euphorigenic properties and liability for dependence have fueled a sustained global opioid pandemic, with profound public health consequences. The proliferation of high-potency synthetic opioids, particularly fentanyl and its analogs, has intensified this pandemic, driving more than 100,000 overdose deaths annually in the United States and sustaining an unprecedented public health crisis [33]. BUP combined with psychosocial support represents evidence-based interventions that reduce illicit opioid use and mortality. Despite strong efficacy in controlled trials, real-world implementation remains limited by structural barriers to access, adherence challenges associated with daily dosing, stigma linked to frequent clinic attendance and missed doses that increase relapse risk [8, 13, 14]. ULA formulations offer a translational strategy to mitigate these constraints by sustaining therapeutic exposure, reducing clinical burden, minimizing “highs and lows” in drug exposure, and improving treatment continuity [22]. This approach is favored by patients and has been validated in other chronic disease settings [34, 35].

Herein, we designed a conformationally restricted BUP prodrug and ion-paired it with pamoic acid to form M6BUP-Pamoate salt, facilitating the creation of a concentrated aqueous-based ULA nanosuspension (NM6BUP-Pamoate). The ULA nanosuspension produced sustained efficacious systemic and brain exposure to BUP for up to six months following a single 45 or 90 mg/kg IM or SC dose administration. Notably, unlike the current monthly BUP in situ depot formulation approaches, NM6BUP-Pamoate leverages an aqueous injectable nanosuspension platform with an established clinical precedent in chronic indications, such as HIV and psychotic disorders, where large-scale clinical studies have demonstrated significant improvements in adherence, treatment durability, and patient satisfaction. Such improvements in BUP’s pharmacology could improve overall clinical uptake and medication-assisted therapy (MAT) efficacy in managing opioid overdose, considering that BUP’s real-world clinical impact remains limited by adherence barriers and misuse potential associated with the existing formulations [36]. The currently approved monthly LA BUP formulations, Sublocade and Brixadi, partially address adherence but are limited by dosing frequency and injection-site reactions [37, 38]. A six-month BUP implant, Probuphine, addressed these limitations [17]; however, its clinical use was discontinued, in part due to major challenges associated with provider uptake and the requirement for surgical procedures, PK variability concerns, implant-related adverse events, and manufacturing challenges. Our reported NM6BUP-Pamoate replicates the sustained-release profile for 6-months without the disadvantages of administration and local site toxicity, positioning NM6BUP-Pamoate as a clinically translatable ULA BUP formulation for major extension in the dosing interval, with the potential to reduce treatment burden and improve long-term outcomes in opioid use disorder.

To develop an ultralong-acting aqueous-based BUP formulation, M6BUP free-base prodrug and its corresponding hydrophobic ion pair, M6BUP-Pamoate, were designed. The ideal water-insoluble prodrug must exist in a solid form to facilitate the development of aqueous-based nanocrystals. Other lipidated ester prodrugs of BUP reported in the literature are inherently oily liquids and are incompatible with established micronization formulation technologies for industrial aqueous suspensions. In contrast, the M6BUP free-base prodrug is a conformationally restricted, water-insoluble, free-flowing powder that releases BUP upon hydrolysis. The prodrug was synthesized using a scalable two-step esterification strategy employing stearoylated aminobenzoic acid, selected based on the safety profiles of stearic acid and 4-aminobenzoic acid breakdown products. Importantly, a comprehensive cell-based functional safety screen encompassing 78 clinically relevant molecular targets revealed no off-target agonist, antagonist, or inhibitory activity, including no inhibition of the hERG potassium channel by the M6BUP prodrug solution. These findings demonstrate the nonclinical safety and reduced risk of adverse reactions associated with M6BUP. Pamoic acid also has a well-established safety record, having been used in approved LA pharmaceutical products [20][30]. Consistent with these precedents, injection-site assessments conducted during both the NM6BUP and NM6BUP-Pamoate PK studies revealed no macroscopic abnormalities following administration. Longitudinal monitoring of body weight and serum chemistry parameters showed no evidence of systemic toxicity in the treated animals compared to the control animals. Histological evaluation of brain tissue sections following administration of a high dose of 90 mg/kg BUP-equivalent demonstrated no associated histopathological alterations, indicating the tolerability of the formulation. By day 182, the injection site tissues showed no histopathological changes. The results provided evidence for the safety of the ULA NM6BUP-Pamoate.

The conversion of NM6BUP to the hydrophobic ion pair NM6BUP-Pamoate substantially increased the formulation drug content from 114.3 mg/mL for the free base to over 300 mg/ml for the prodrug ion pair suspension. The enhanced drug concentration within the salt-based formulation has the potential to significantly reduce the injection volume for human dosing, which is a key determinant of patient comfort and injection-site tolerability [40]. Reducing injection volumes is particularly relevant, given that injection-site reactions have been identified as a limiting factor in the uptake of currently approved monthly BUP formulations, such as Sublocade [37]. Additionally, the use of N-methyl-2-pyrrolidone (NMP) solvent in Sublocade was associated with pronounced injection-site discomfort, likely due to organic solvent concentrations and depot phase inversion kinetics, necessitating adjunctive pain-mitigation strategies and contributing to patient and clinic hesitancy [15]. In contrast, NM6BUP-Pamoate is formulated as an aqueous nanosuspension using PEG-3350 and Tween-20, excipients that are also employed in clinically approved long-acting nanosuspension products and have well-established safety profiles [41]. A ULA well-tolerated NM6BUP-Pamoate formulation has the potential to dramatically improve compliance, efficacy, and patient satisfaction.

The extended PK profiles observed for both NM6BUP and NM6BUP-Pamoate are closely linked to their physicochemical properties, particularly enhanced hydrophobicity, slow prodrug dissolution, conversion kinetics, and tissue-level sequestration of the prodrug. Chemical modification of BUP with a benzylated long-chain fatty-acid ester markedly enhanced the compound’s hydrophobicity relative to that of the parent BUP, as reflected by a substantial shift in partitioning behavior, with aqueous solubility reduced from approximately 1.2 μg/mL for BUP to 0.21 μg/mL for M6BUP, while maintaining high octanol solubility. The subsequent hydrophobic ion pair, M6BUP-Pamoate exhibited enhanced lipophilicity with an octanol solubility of 165 mg/mL, representing a marked increase in the salt relative to both the BUP and free base M6BUP prodrugs. Enhanced lipophilicity is expected to slow prodrug dissolution and prolong its half-life. In plasma hydrolysis studies, both rat and human matrices demonstrated gradual conversion of M6BUP to BUP over 48 h, with the majority of prodrug remaining intact and only modest accumulation of released BUP, indicating that ester bond cleavage proceeds in a controlled and sustained manner upon prodrug dissolution. This controlled hydrolysis profile supports a dissolution and absorption-limited PK paradigm in which systemic exposure to BUP is governed by the rate of prodrug absorption and dissolution. Sequestration of the formulation within phagocytic cells acts as an additive for the ULA PK profile [42]. In vitro studies in MDMs demonstrated efficient uptake of NM6BUP, with substantial intracellular accumulation of both the M6BUP prodrug and released BUP over time, followed by prolonged intracellular retention. These findings are consistent with previous reports describing macrophage-mediated drug absorption and depots for LA injectable ARV formulations [26, 29]. In vitro retention studies further showed that intracellular drug levels remained measurable for weeks following brief exposure, indicating that macrophages can serve as secondary reservoirs that slowly release active drugs back into the surrounding tissues. This cellular sequestration mechanism is further supported by TEM imaging, which revealed discrete formulation particles localized within intracellular compartments, consistent with phagolysosomal structures.

The ULA PK profile observed following NM6BUP-Pamoate administration is consistent with the formation of a stable, long-lasting drug depot that supports sustained, steady systemic BUP exposure without dose-dependent early burst release. Increasing the IM dose did not result in a proportional increase in the early plasma concentrations. Median day 1 BUP levels were comparable between the 45 and 90 mg/kg IM groups (8.1 (4.2–22.0) ng/mL vs. 11.5 (7.3–17.1) ng/mL, respectively), with similarly overlapping concentrations observed on day 3. The timing and magnitude of peak exposure remained largely unchanged, with no statistically significant differences despite a two-fold increase in dose (T_max_ = 14 days for both 45 and 90 mg/kg, C_max_ 30.7 ± 5.7 ng/mL vs. 43.8 ± 3.2 ng/mL; not significant by ANOVA analysis). Early systemic exposure appears to be buffered against dose escalation, suggesting that initial systemic drug exposure is potentially governed by the rate of prodrug absorption and dissolution rather than by the amount of drug administered. The administered concentrated formulation is expected to undergo slow absorption due to the decreased surface area-to-volume ratio of the depot. Notably, the cumulative systemic exposure increased with dose. Total exposure, as measured by AUC_0–∞_, increased from 815 ± 50.0 to 1271 ± 87.0 ng· day/mL for 45 and 90 mg/kg IM dosing, respectively. This divergence between the controlled early burst release and cumulative exposure suggests that additional drug mass is retained within the depot and released gradually over time, thereby extending the duration and magnitude of the pseudo-plateau phase of drug exposure without introducing burst-related liabilities.

The dissolution and absorption-limited PK profile of NM6BUP-Pamoate supports the potential for dose escalation of the ULA formulation when greater or more sustained exposure is clinically required. Importantly, the PK profile of the ULA formulation was conserved across the routes of administration. A single SC dose of 45 mg/kg produced a similarly prolonged exposure profile (median T_max_ 10.5 (7–14) days) and was associated with longer MRT times than IM administration. The convergence of the IM and SC profiles supports a shared depot-driven mechanism, wherein local drug dissolution and absorption, rather than route-specific factors, dictate systemic exposure. Sustained systemic BUP exposure also translated into even more pronounced CNS drug delivery, where at day 182, BUP concentrations in the central nervous system were 8–12-fold higher than the corresponding plasma levels across the IM-low, IM-high, and SC-low groups, supporting sustained u opioid receptor engagement in the brain.

Beyond pharmacology, the process chemistry of M6BUP was developed, and the laboratory synthetic route was optimized to multidecagram manufacturing batches delivering ≥ 99% pure prodrug, demonstrating scale-up feasibility for potential clinical translation. Taken together, the development of NM6BUP-Pamoate represents a rational evolution of an organic solvent-free ULA BUP formulation design that merges prodrug chemistry and hydrophobic ion-pair approaches. Even though IM injections may be well tolerated in underweight OUD patients who have limited adipose tissue layer, Sublocade^®^ and Brixadi^®^ are designed for SC administration. Flexible IM and SC dosing options for NM6BUP-Pamoate could improve patient and provider satisfaction. A well-tolerated every-six-months NM6BUP-Pamoate formulation has the potential to improve patient convenience and adherence to medication and ultimately reduce opioid overdose fatalities.

## 4. METHODS AND MATERIALS

### 4.1. Materials

Reagents were obtained from commercial sources and used directly, except where noted. BUP was purchased from Veranova Macfarlan Smith, Ltd. (Edinburgh, UK). 4-amino benzoic acid, stearoyl chloride, tetrahydrofuran (THF), pyridine, polysorbate 20 (Tween 20), polyethylene glycol-3350 (PEG-3350), ciprofloxacin, 3-(4,5-dimethylthiazol-2-yl)-2,5-diphenyltetrazolium bromide (MTT), dimethyl sulfoxide (DMSO), 1-octanol, paraformaldehyde (PFA), 3,3’-diaminobenzidine (DAB), and 1-(3-dimethylaminopropyl)-3-ethylcarbodiimide hydrochloride (EDC·HCl) were purchased from Sigma-Aldrich (St. Louis, MO, USA). Diethyl ether, acetonitrile (ACN), methanol, Optima-grade water, ethyl acetate, hexanes, dichloromethane (DCM), Dulbecco’s Modified Eagle’s Medium (DMEM), phosphate-buffered saline (PBS), gentamicin, L-glutamine, potassium phosphate monobasic (KH_2_PO4), bovine serum albumin (BSA), and Triton X-100 were purchased from Thermo Fisher Scientific/Gibco (Waltham, MA, USA). Cell culture-grade water (endotoxin-free) was purchased from Cytiva (Logan, UT, USA). Heat-inactivated pooled human serum was purchased from Innovative Biologics (Herndon, VA, USA). The Limulus Amebocyte Lysate Pyrogent-500 endotoxin test kit was purchased from Lonza. The lancets were purchased from MEDIpoint Inc. All MS-grade solvents were purchased from Fisher Scientific. Sprague–Dawley rats (270–300 g, approximately 8 weeks of age) were sourced from SASCO (Wilmington, MA, USA) and acclimatized prior to study initiation.

### 4.2. Methods

#### 4.2.1. Study approval

The University of Nebraska Medical Center Institutional Animal Care and Use Committee (IACUC) approved all PK studies involving laboratory animals according to the standards incorporated in the Guide for the Care and Use of Laboratory Animals (UNMC IACUC protocol number: 23-056-09-EP). Human peripheral blood monocyte isolation and purification from HIV-1/2- and HBV-seronegative participants were performed according to the UNMC Institutional Review Board (IRB) guidelines, with informed consent.

#### 4.2.2. Synthesis and characterization of M6BUP free base and M6BUP-Pamoate salt

To synthesize the linker, one equivalent of 4-aminobenzoic acid (4 g scale) was dissolved in 110 mL of tetrahydrofuran (THF) and cooled in an ice bath. This was followed by the addition of 30 mL of 2 M NaOH and 1.6 eq. of stearoyl chloride. The resulting suspension was heated at 40°C and stirred overnight (approximately 16 h). After the reaction was complete, THF was evaporated using a rotary evaporator. The aqueous phase was suspended in 200 mL of Milli-Q water, cooled on ice, and acidified to pH 1 with concentrated HCl (12 M). The resulting precipitate was collected by filtration and washed sequentially with 200 mL of water and 200 mL ether. The collected solid was then resuspended in a mixture of 200 mL acetone and 150 mL water, stirred at room temperature for approximately 10 min, and filtered again. The solid was further washed with 200 mL of water, followed by 200 mL of ether, and dried under high vacuum. The final product was obtained as an off-white powder with a chemical yield of over 90%. Structural confirmation was performed using NMR spectroscopy in deuterated DMSO. The linker was used in subsequent reactions without purification.

For the synthesis of M6BUP, one equivalent of BUP (3 g scale) was dissolved in 30 mL of anhydrous pyridine under an argon atmosphere and cooled in an ice bath. Subsequently, 2.2 equivalents of 4-stearamidobenzoic acid and 4 equivalents of 1-(3-dimethylaminopropyl)-3-ethylcarbodiimide hydrochloride (EDC·HCl) were added. The reaction mixture was heated at 70°C and stirred overnight (~ 16 h) under argon atmosphere. Thin-layer chromatography (TLC) on silica gel plates using a 2:1 hexane/ethyl acetate (H/EA) solvent system was used to monitor the reaction progress of the reaction. As BUP is not strongly UV-active, TLC plates were visualized using potassium permanganate (KMnO_4_) staining. Aliquots were periodically withdrawn and compared with BUP and linker standards. Upon completion, the reaction mixture was concentrated using a rotary evaporator, diluted with 100 mL of dichloromethane (DCM), and transferred to a separatory funnel. The organic phase was washed with brine and dried over anhydrous sodium sulfate (Na_2_SO_4_). The solvent was evaporated, and the desired compound was purified by column chromatography. Silica gel (180 g; particle size 60–200 μm) was packed into a column (40.0 mm ID × 457 mm L) to a bed height of 300 mm using a 4:1 hexane/ethyl acetate solvent system. The column was initially eluted with approximately 500 mL of 4:1 hexane/ethyl acetate, followed by 100 mL of 2:1 hexane/ethyl acetate solution. Under these conditions, the UV-active pure M6BUP began to elute and continued to elute over approximately 400 mL of solvent. The appropriate fractions were collected and concentrated under reduced pressure. The product was further dried under high vacuum for ~ 1 h, washed with ether, and placed on ice to induce precipitation. The solid was isolated by filtration and dried to produce the final purified compound.

Scaling of M6BUP production to the multidecagram scale was conducted in an independent contract manufacturing facility, Veranova L.P. (Devens, MA, USA) (Project ID: P4499/01). Briefly, the reactions were performed in a 2 L jacketed glass reactor (Radleys) under nitrogen atmosphere. Pyridine (943 g, 960 mL, 93.9 Eq, 11.9 mol) was added to BUP.HCl (64.0 g, 1 Eq, 127 mmol) with stirring (200–300 rpm) until a clear solution was formed. The mixture was cooled to 5°C, and 4-stearamidobenzoic acid (61.5 g, 1.2 eq, 152 mmol) was added in a single portion, followed by EDC·HCl (73.0 g, 3 eq, 381 mmol). The reaction mixture was heated to 60°C, at which point it became homogeneous. The reaction progress was monitored by HPLC, and supplementary additions of EDC·HCl and linker were made over ~ 4 days to drive the conversion to completion. At the end of the reaction, the reaction mixture was concentrated under reduced pressure to produce a red oily mixture. The reaction mixture was then partitioned between ethyl acetate (1 L) and 20% w/w brine solution (2 L) and stirred at 20°C for 30 min. The biphasic mixture formed a persistent emulsion, which was resolved by warming to 50°C and performing sequential ethyl acetate extractions. The combined organic layers were dried over MgSO_4_, filtered, and concentrated to produce a crude material. The crude material was purified by flash chromatography on silica gel using seven columns (six crude-load; one mixed-fraction), each packed with ~ 650 g silica (~ 25 w/w relative to crude). Elution used ~ 80 L of 4:1 heptane: ethyl acetate, then 1:1 heptane: ethyl acetate. A total of 195 fractions (~ 250 mL each) were collected, and the fractions that met an internal purity specification of ≥ 99.5% (HPLC–DAD) were combined, polish-filtered (GF/D), and concentrated. The process yielded 100 g of damp solid (92.3%) and 64 g of dried M6BUP (59% yield).

To synthesize the M6BUP-Pamoate ion pair, 7.03 mmol of pure M6BUP free base powder (6 g, 2 equivalents) was dissolved in 35 mL of DMSO. To this solution, 3.516 mmol of pamoic acid (1.406 g, 97% purity, 1 eq.) was added. The reaction mixture was then heated to 70°C and stirred for approximately 1 h under an argon atmosphere. After 1-h, the resultant solution was added to 600 mL of Milli-Q water with stirring to precipitate the salt. The salt suspension was collected by filtration (suction filtration). The solid was then washed twice with 350 mL of water. The resultant salt product was dried using a high-vacuum pump for 16h. Structural confirmation and physicochemical characterization by NMR, FT-IR, XRD, and DSC were performed following the procedures and instrumentation outlined in Section 2.2.2.

The large-scale production feasibility of M6BUP-Pamoate was evaluated at an independent contract manufacturing facility. Briefly, M6BUP (3.01 g) was added to an EasyMax vessel (50 ml), followed by the addition of DMSO (6 vol; 18 mL). The mixture was stirred at 350 rpm and heated to 60°C to form an emulsion within 5 min. Pamoic acid (0.5 equiv; 682.7 mg) was then added, and stirring at 60°C was continued for an additional 5 min to obtain a clear yellow solution. The reaction mixture was then transferred via pipette into water (17 vol; 51 mL) to generate a suspension. The suspension was stirred at room temperature for 20 min, cooled to 5°C, and held overnight. The resulting solids were collected by vacuum filtration using a glass Buchner funnel fitted with a 0.45 μm nylon filter paper. The wet cake was dried under suction for 10 min and then dried in vacuum at room temperature for 1 h. The dried material was then analyzed using NMR and FT-IR.

All chemical reactions were performed under an inert argon atmosphere, unless otherwise stated. Structural confirmation of the final products was achieved using proton and carbon nuclear magnetic resonance spectroscopy (^1^H and ^13^C NMR) on a Bruker Avance III HD spectrometer (500 MHz, 11.7 T; Billerica, MA, USA). The functional group integrity and chemical bonding features were further validated using Fourier-transform infrared (FT-IR) spectroscopy with a PerkinElmer Spectrum Two instrument equipped with a universal attenuated total reflectance (ATR) accessory (Waltham, MA, USA). The molecular masses of the purified compounds were determined using direct-infusion mass spectrometry on a Thermo Fisher Exploris 480 mass spectrometer. The solid-state properties and crystallographic features of the synthesized materials were examined using powder X-ray diffraction (PXRD) with a PANalytical Empyrean diffractometer (PANalytical Inc., Westborough, MA, USA). Diffraction data were collected over a 2θ range of 2–50° using Cu Kα radiation (λ = 1.5418 Å) operated at 40 kV and 45 mA.

#### 4.2.3. Analysis of M6BUP by UPLC-MS/MS

UPLC-MS/MS quantitation of BUP and M6BUP was performed using a Waters TQ-XS coupled with a Waters Acquity Premier UPLC system. Chromatographic separation of BUP and M6BUP was achieved on a Waters ACQUITY UPLC BEH RP18 column (1.7 μm, 2.1 mm × 50 mm) using 7 min gradients of mobile phase A (0.1% formic acid (FA) in H_2_0) and mobile phase B (0.1% FA in Acetonitrile (ACN)) at a flow rate of 0.7 ml/min, with a column temperature of 45°C. During the first 0.5 min, mobile phase B was held at 20%, followed by a linear gradient from 20% B to 80% B between 0.5 and 4.5 min. Subsequently, a linear gradient from 80% B to 95% B was applied from 4.5 to 5 min. Mobile phase B was held at 95% from 5 to 6 min and then returned to its original condition of 20% B at 6.5 min, where it was held for 0.5 min for equilibration. The mass spectrometry conditions and multiple reaction monitoring (MRM) transitions are detailed in the following table: Spectra were analyzed and quantified using Waters MassLynx V4.2 software (Milford, MA, USA). All quantitation was determined using analyte peak area to internal standard peak area ratios, using the MRM transitions of BUP, NM6BUP, and BUP-d4, and M4RPV: 468.52 > 396.18, 853.7 > 386.26, 472.51 > 400.19, 623.51 > 411.17, respectively.

Plasma samples for LC–MS/MS analysis on the TQXS system were prepared using a liquid–liquid extraction procedure. Briefly, 50 μL of the samples or plasma containing the spiking standard was transferred into a 1.6 mL microcentrifuge tube, followed by the addition of 15 μL of internal standard (IS) solution (15 ng/mL BUP-d4) and brief vortexing. The samples were then made basic by adding 15 μL of NH_4_OH and vortexed again to ensure proper mixing of the solution. Subsequently, 210 μL of ethyl acetate and 210 μL of hexane were added, and the tubes were placed on a horizontal shaker for 30 min. Samples were subsequently centrifuged for 8 min at 20,000 × g, after which the tubes were placed on dry ice with acetonitrile to freeze the aqueous layer. Once frozen, 340 μL of the organic upper layer was carefully transferred to a clean microcentrifuge tube. The remaining aqueous layer underwent a second extraction using fresh ethyl acetate:hexane (1:1, v/v), followed by another 30-minute shake and centrifugation at 20,000 × g for 8 min. The resulting organic phase was combined with the initial extract and evaporated to dryness in a speed vacuum concentrator for approximately 2 h. The dried residue was reconstituted in 100 μL of 50:50 (v/v) methanol:water and vortexed thoroughly to achieve complete dissolution of the sample. Samples were then filtered through a standard 0.2 μm filter plate, and the filtrate was collected in LC–MS vials or a 96-well plate for analysis. Finally, 10 μL of each processed sample was injected onto the TQXS triple-quadrupole mass spectrometer using the validated LC gradient, mobile phases, and MRM transitions for the analyte and internal standard.

#### 4.2.4. UPLC-UV/Vis quantitation of M6BUP

UPLC-UV detection for M6BUP was performed using a Waters Acquity H-Class UPLC system equipped with a TUV detector (Waters Corp., Milford, MA) and controlled by Empower 3 software (Version 7.40.00.00). Mobile phase A consisted of 0.1% formic acid in LC-MS-grade water, and mobile phase B consisted of 0.1% formic acid in LC-MS-grade acetonitrile. The needle-wash solution was composed of 50:30:20 methanol:water:acetone. Chromatographic separation was achieved using an Acquity UPLC BEH C18 column (1.7 μm, 2.1 × 50 mm; Waters PN 186002350) fitted with an Acquity UPLC BEH C18 1.7 μm Vanguard^™^ pre-column. The detection was performed at 291 nm using a TUV detector. Spectral data were processed and quantified using Empower 3, with drug concentrations determined by comparing the sample peak areas to the standard peak-area ratios. Calibration curves were generated by plotting the peak area versus the nominal analyte concentration and applying a linear regression model. For formulation quantitation, samples were first diluted 100-fold by adding 10 μL of formulation to 990 μL of fresh HPLC-grade DMSO in triplicate, followed by 8-minute water-bath sonication with an ice pack to prevent temperature elevation. After brief vortexing, the samples were further diluted 10-, 100-, or 1000-fold, depending on the expected drug concentration range (standard curve: 200–1.5625 μg/mL) in HPLC-grade MeOH, and vortexed again. Samples were centrifuged at 14,000 rpm for 10 min at 4°C, and 100 μL of each supernatant was transferred into a 96-well plate. The plates were sealed with a rubber mat to prevent cross-contamination and loaded onto the UPLC-UV system for analysis.

#### 4.2.5. Octanol and Aqueous solubility assessment

The hydrophobicity and lipophilicity of the prodrug and BUP were determined using a shake flask approach. Briefly, an excess amount of the drug was added to either purified water or 1-octanol and agitated continuously at ambient temperature for 24 h to ensure saturation equilibrium. Following incubation, the suspensions were pelleted by centrifugation at 20,000 × g for 10 min to remove any undissolved material. The resulting supernatants with dissolved drugs were collected for quantitative analysis using UPLC-MS/MS.

#### 4.2.6. Isolation and cultivation of human monocyte-derived macrophages (MDM)

Human monocytes from HIV-1/2-and hepatitis B-seronegative donors were acquired through leukapheresis and subsequently purified by countercurrent centrifugal elutriation. Following isolation, the monocytes were cultured according to the established procedures developed in our laboratory [27]. Once differentiated, MDMs were used to assess drug particle uptake and retention.

#### 4.2.7. Cellular vitality assays

The effects of free-drug exposure on the metabolic activity of monocyte-derived macrophages (MDMs) were evaluated using a colorimetric tetrazolium-based viability assay using the 3-(4,5-dimethylthiazol-2-yl)-2,5-diphenyltetrazolium bromide (MTT) assay, as described in our previously published research papers [27]. In brief, MDMs were treated with increasing concentrations of the test compounds (1.95–500 μM) and incubated for 24 h under standard culture conditions. The parallel control groups received either vehicle alone or no treatment. At the end of the exposure period, the cells were gently rinsed to remove residual compounds and incubated with MTT reagent prepared in complete culture medium for 45 min. The reaction medium was then aspirated, and dimethyl sulfoxide (DMSO; 200 μL per well) was added to dissolve the formazan. Absorbance was recorded at 490 nm using a SpectraMax M3 microplate reader (Molecular Devices, LLC, Sunnyvale, CA, USA), and quantitative analysis was performed using SoftMax Pro software (version 6.2).

#### 4.2.8. Prodrug metabolism in biorelevant media

The susceptibility of M6BUP metabolism to BUP through enzymatic pathways was examined in biologically relevant media. Hydrolysis experiments were performed using plasma collected from rats and humans. Prior to the initiation of the reaction, the plasma was equilibrated at 37°C. M6BUP, prepared as a concentrated stock in methanol (1% v/v), was introduced into the pre-warmed matrices to achieve a final concentration of 1 μM. The incubations were maintained at 37°C for up to 24 h to allow for the time-dependent conversion of the prodrug. At predetermined intervals, aliquots were withdrawn and immediately quenched with ice-cold aqueous methanol (80%, v/v) containing 0.1% formic acid to terminate enzymatic activity and precipitate the proteins. Following mixing, the samples were pelleted by centrifugation at 16,000 × g for 10 min, and the resulting supernatants were subjected to quantitative analysis of both M6BUP and released BUP using a validated UPLC–MS/MS method.

#### 4.2.9. In-vitro off-target Selectivity Assay of M6BUP and BUP

Off-target pharmacology for M6BUP and BUP was assessed using a 78-target safety panel (SAFETYscan E/IC_50_ ELECT service offered by an independent contract research organization, Eurofins Discovery, San Diego, CA, USA), which encompasses a broad range of human-relevant receptors, ion channels, transporters, kinases, and enzymes. The panel integrates multiple assay platforms, including PathHunter^®^ enzyme fragment complementation (EFC) assays for GPCR function, FLIPR-based cellular assays for ion channel and calcium flux readouts, KINOMEscan^®^ binding assays for kinase selectivity profiling, and complementary enzymatic assays. The results were reported as the percentage activity relative to the target-specific positive and negative controls. Functional responses exceeding 50% in either direction were predefined as indicative of meaningful agonist/antagonist activity. To further characterize the potential opioid receptor activity, an expanded GPCR screen offered by Eurofins Discovery was performed. cAMP signaling was assessed using HitHunter^®^ EFC technology, employing β-galactosidase complementation as the reporter system. Potential positive allosteric modulation (PAM) at μ-, δ-, and κ-opioid receptors was evaluated using the HitHunter^®^ cAMP XS+ assay, configured for allosteric detection.

#### 4.2.10. Free base prodrug and salt formulation process and characterization

Surfactant-stabilized nanosuspensions of the free-base M6BUP prodrug were generated via high-pressure homogenization. Drug powders were first dispersed in an aqueous surfactant solution prepared in phosphate-buffered saline (PBS; 11.9 mM potassium phosphate monobasic, 137 mM sodium chloride, and 2.7 mM potassium chloride) and allowed to equilibrate under gentle agitation overnight. The particle size reduction and homogenization of the pre-dispersed suspensions were performed using a high-pressure homogenizer operated at 20,000 psi. Particle size reduction was monitored intermittently by collecting samples during homogenization, which was discontinued once an average hydrodynamic diameter of 300–450 nm was achieved. The free-base nanosuspensions were then collected and lyophilized. Prior to characterization, the lyophilized formulations were reconstituted in PBS to the required drug concentrations.

M6BUP-Pamoate salt was produced to enable the production of a concentrated ready-to-use nanosuspension. To prepare the prodrug ion pair formulations, M6BUP-Pamoate powders were first dispersed in aqueous surfactant solutions of tween-20 and PEG-3350 in phosphate-buffered saline (PBS; 11.9 mM potassium phosphate monobasic, 137 mM sodium chloride, and 2.7 mM potassium chloride) at ratios of 7:1:1 (Drug: Tween-20: PEG3350 w/w). The microsuspension was gently agitated overnight to promote uniform wetting and equilibration of the drug. Particle size reduction was subsequently achieved by processing the suspension using a high-pressure homogenizer operated at 20,000 psi. Aliquots were withdrawn periodically during homogenization to monitor particle parameters until the mean hydrodynamic diameter approached ~ 300 nm. The resulting nanosuspension was collected and characterized. The long-term physicochemical stability of the NM6BUP-Pamoate formulation was assessed under storage at room temperature and 4°C for up to 182 days. The stability parameters included hydrodynamic particle size, PDI, zeta potential, visual appearance, and drug content. The particle size distribution and surface charge were measured using dynamic light scattering. The drug content within the formulation was quantified using UPLC–MS/MS following dissolution in methanol. The endotoxin burden was determined using a kinetic chromogenic assay (Endosafe nexgen-PTS, Charles River), and syringeability testing confirmed that the formulation could be readily administered through a 28-gauge needle.

Physicochemical stability was evaluated under storage at ambient temperature and 4°C, including the assessment of particle size, polydispersity, surface charge, formulation appearance, and drug content. Dynamic light scattering was employed to determine the hydrodynamic diameter, polydispersity index, and zeta potential. The drug content within the formulation was quantified using UPLC–MS/MS following dissolution in methanol. Endotoxin levels were assessed using a Charles River Endosafe nexgen-PTS system (Charles River, USA), and injectability testing confirmed that all formulations were readily deliverable through a 28-gauge needle.

#### 4.2.11. Transmission electron microscopy

Transmission electron microscopy was used to visualize intracellular nanoparticle localization in MDMs after 8 h of drug treatment. Cells were washed, scraped into PBS, pelleted at 3000 RPM for 8 min at room temperature, and fixed in a solution of 2% glutaraldehyde and 2% paraformaldehyde in 0.1 M Sorenson’s phosphate buffer (pH 6.2). A drop of the fixed cell suspension was placed on a formvar/silicon monoxide 200 mesh copper grid, allowed to settle for 2 min, the excess solution was wicked off, and the grid was allowed to dry. A drop of NanoVan vanadium negative stain was placed on the grid for 1 min, then wicked away and allowed to dry. Grids were examined using an FEI Tecnai G2 Spirit TWIN transmission electron microscope (Hillsboro, OR, USA) operated at 80 kV, and images were acquired digitally using an AMT digital imaging system (Woburn, MA, USA).

#### 4.2.12. Uptake and retention of NM6BUP in MDMs

*In vitro* cellular internalization of nanoparticles and long-term intracellular retention of NM6BUP in MDMs were evaluated using established *in vitro* MDMs models adapted from previously validated laboratory protocols [27]. For the uptake experiments, MDMs were incubated with NM6BUP nanoformulations at a concentration of 25 μM for 24 h. For long-term intracellular retention assessments, cells were initially exposed to the same nanoformulations (25 μM) for 8 h, after which the treatment medium was replaced with a drug-free culture medium. The cells were then maintained for up to 20 days to assess intracellular retention following drug withdrawal. At predefined time points, macrophages were harvested and lysed using methanol to extract intracellular drug and prodrug species. The quantitative determination of NM6BUP and BUP levels was performed using UPLC-MS/MS.

#### 4.2.13. NM6BUP free base formulation PK and toxicology studies in SD rats

PK and biodistribution (BD) evaluations of the NM6BUP free-base formulations were conducted following IM administration in SD rats (8 weeks of age; SASCO, Wilmington, MA, USA). The free base formulations were prepared, lyophilized, and subsequently reconstituted in phosphate-buffered saline (PBS) to a final concentration of 114.3 mg/mL, prior to dosing. The formulations were characterized by particle size, PDI, and zeta potential to ensure consistency across the treatment groups. Male SD rats of comparable age and body weight were allocated to two treatment cohorts receiving a single IM dose equivalent to either 45 or 90 mg/kg BUP. For the 45 mg/kg dose group (n = 8), injections were administered into the left caudal thigh muscle at a volume of 0.718 μL/g of body weight. Animals in the 90 mg/kg group (n = 4) received bilateral caudal thigh injections of a total volume of 1.437 μL/g body weight. All injections were performed using 1 mL syringes with 25-gauge needles. Serial blood samples were collected on days 1, 3, and 7 post-dose and at weekly intervals thereafter for the duration of the experiment. Blood was obtained via tail vein sampling into heparinized tubes and processed by centrifugation at 2,000 × g for 8 min to isolate plasma. Plasma samples were stored at − 70°C until quantitative drug analysis. Drug analysis was performed as described in Section 4.2.4. Non-compartmental pharmacokinetic analysis of plasma drug concentrations was performed using Phoenix WinNonlin (version 8.3.3.33; Certara).

To assess long-term biodistribution, animals in the 45 mg/kg cohort were assigned to interim and terminal necropsy time points for analysis. Four animals were randomly selected and euthanized on day 56 post-dose, and the remaining animals were maintained until day 182. Animals in the 90 mg/kg cohort were maintained until Day 182. At each necropsy, tissues were collected and subdivided for quantitative drug analysis and histopathological evaluation. For histological analysis, the tissues were fixed in buffered formalin, paraffin-embedded, sectioned to a thickness of 5 μm, and stained with hematoxylin and eosin. Tissue sections were imaged using Echo Revolution Microscope (San Diego, CA, USA) and evaluated by an independent board-certified pathologist who was blinded to the treatment groups. Systemic biochemical parameters were measured using an automated clinical chemistry analyzer (VetScan VS-2; Abaxis Veterinary Diagnostics), and Serum chemistry values from NM6BUP-treated animals were compared with those from a separate group of age-matched untreated Sprague–Dawley rats used as reference controls.

Throughout the study period, the animals were maintained under standardized housing conditions, including a controlled 12 h light/dark cycle, ambient temperature maintained targeted between 20 and 24°C, and relative humidity between 30% and 70%. The rats received a sterilized laboratory rodent diet (Teklad LM-485 Mouse/Rat Diet 7012; Harlan Teklad, Madison, WI, USA) ad libitum and were allowed unrestricted access to acidified water. The animals were observed throughout the study for overall health, behavioral changes, and injection-site tolerance. Body weights were recorded before dosing and at each scheduled blood-collection time point throughout the study as a longitudinal measure of systemic tolerability. The non-fasted animals were anesthetized with inhaled isoflurane during blood sampling. The animals were euthanized by isoflurane inhalation followed by cervical dislocation.

#### 4.2.14. PK and toxicology study of NM6BUP-Pamoate ion pair formulations in SD rats

For NM6BUP-Pamoate plasma pharmacokinetics, both IM and SC administrations were assessed in male Sprague–Dawley (SD) rats (8 weeks of age). NM6BUP-Pamoate formulation was produced as a ready-to-use suspension at a drug concentration of 245 mg/ml for rat PK studies. Prior to dosing, the formulations were characterized based on particle size, PDI, and zeta potential to ensure consistency across the treatment groups. Animal age and body weights were similar to those of SD rats used for the NM6BUP free base PK study and randomly divided into 5 groups. Animals receiving the 45 mg/kg BUP equivalent dose were administered either IM (n = 4, group 1) into the left caudal thigh muscle or SC (n = 4, group 2) into the dorsal scruff region at an injection volume of 0.335 μL/g body weight. An additional cohort received a higher IM dose of 90 mg/kg BUP equivalent (n = 4, group 3) at an injection volume of 0.669 μL/g body weight, administered into the left caudal thigh muscle. Following administration, serial blood samples were collected on days 1, 3, and 7 post-dose and subsequently at weekly intervals for the duration of the PK studies. The animals were sacrificed after day 182 of blood collection.

An additional NM6BUP-Pamoate formulation was produced at a higher drug concentration (302 mg/ml) without lyophilization and reconstitution. NM6BUP_high concentration_ was also assessed in SD rats after a single 45 or 90 mg/kg BUP-equivalent IM administration. Animals in the 45 mg/kg BUP equivalent dose group (n = 4, group 4) received injections into the left caudal thigh muscle at a volume of 0.271 μL/g body weight, whereas the 90 mg/kg BUP equivalent cohort (n = 4, group 5) received IM injections at a volume of 0.543 μL/g body weight. All injections were performed using 1 mL syringes fitted with 25-gauge needle. Following administration, serial blood samples were collected on days 1, 3, and 7 post-dose and subsequently at weekly intervals for the duration of the PK studies. Plasma was isolated and drug concentration were determined by UPLC-MS/MS. Non-compartmental pharmacokinetic analyses were performed using Phoenix WinNonlin software (version 8.3.3.33). Animals in Groups 4 and 5 were sacrificed after 126 and 154 days, respectively. At each necropsy, tissues were collected and subdivided for quantitative drug analysis, histopathological evaluation, and serum chemistry assessment. The procedures for histological assessment and serum chemistry were as described for NM6BUP PK. Anesthetization, blood sampling procedures, necropsy procedures, animal welfare and maintenance, and food supply were similar to those in the NM6BUP PK study 4.2.13.

#### 4.2.15. Statistics

All quantitative data processing and statistical evaluations were performed using Microsoft Excel (version 16.49; Microsoft Corp., Redmond, WA, USA) and GraphPad Prism (version 9.3; GraphPad Software, La Jolla, CA, USA). The experimental results are presented as mean ± standard error of the mean (SEM). Statistical significance was set at P < 0.05. Data points classified as extreme outliers—defined as measurements exceeding three times the group mean and falling outside the 99% confidence limits—were omitted from the further analysis. Chemical synthesis, physicochemical characterization, and formulation development procedures were independently reproduced a minimum of five times to confirm the methodological robustness. Enzymatic and chemical hydrolysis assays, as well as all in vitro experiments, were conducted using at least three independent biological replicates and repeated on no fewer than two separate occasions, yielding consistent and reproducible outcomes. The formulation manufacturing and physical characterization of the nanoformulations were successfully reproduced at least five times. The solubility of the drugs was compared using a two-tailed Welch’s t-test with α = 0.05. Longitudinal plasma BUP levels among the different groups were compared using one-way ANOVA followed by Tukey’s multiple comparisons. Non-compartmental PK parameters derived from plasma BUP concentrations following nanoformulation dosing were compared using either an unpaired two-tailed t-test with Welch’s correction or an ordinary one-way ANOVA with Tukey’s multiple-comparisons correction (α = 0.05).

## Supplementary Material

This is a list of supplementary files associated with this preprint. Click to download.

- SupplementaryInformationSultanaetal.docx
- ReportingSummary.pdf

## Figures and Tables

**Figure 1:**
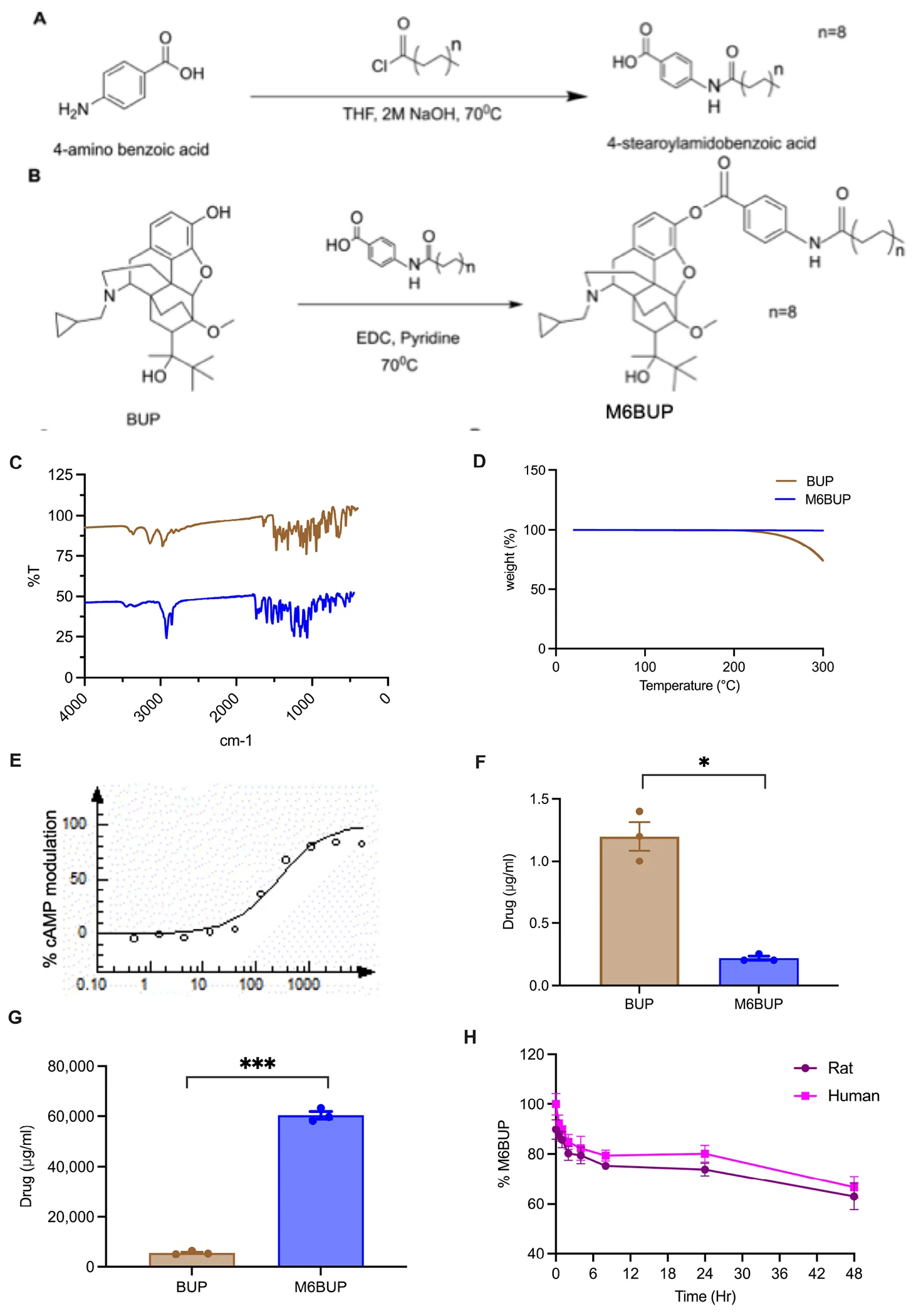
M6BUP free base synthesis and characterization. **A** Schematic illustration of M6BUP prodrug promoiety synthesis, where 4-aminobenzoic acid was reacted with stearoyl chloride in the presence of NaOH base at 70 °C. **B** Synthesis of M6BUP via carbodiimide-mediated coupling of 4-stearoylaminobenzoic acid to buprenorphine (BUP) using EDC as the coupling reagent. **C** Overlay of Fourier-transform infrared (FT-IR) spectra of BUP and M6BUP, showing characteristic methylene stretching bands at 2854 and 2918 cm^−1^ corresponding to the lipid promoiety, and an ester carbonyl stretch at 1751 cm^−1^ confirming prodrug formation. **D** Thermogravimetric analysis (TGA) comparing the thermal stability of BUP and M6BUP. **E** The prodrug M6BUP did not exhibit agonism, antagonism, inhibition or activation of any target except for agonism of MOR (EC50 = 0.256 μM). **F-G** Aqueous and octanol solubility of BUP and M6BUP quantified by LC-MS/MS. Data are presented as mean ± SEM (n = 3 independent replicates). Statistical comparisons were performed using two-tailed Welch’s t-test (**P < 0.05). **H** In vitro stability of M6BUP in rat and human plasma following incubation with 1 μM M6BUP at 37 °C for up to 48 h. Data are expressed as mean ± SEM (n = 3 independent replicates). Panels A–D were reproduced at least five times and panels E–H at least twice with comparable results. Source data are provided in the Figshare database under Digital Object Identifier (DOI) code (10.6084/m9.figshare.34052907).

**Figure 2:**
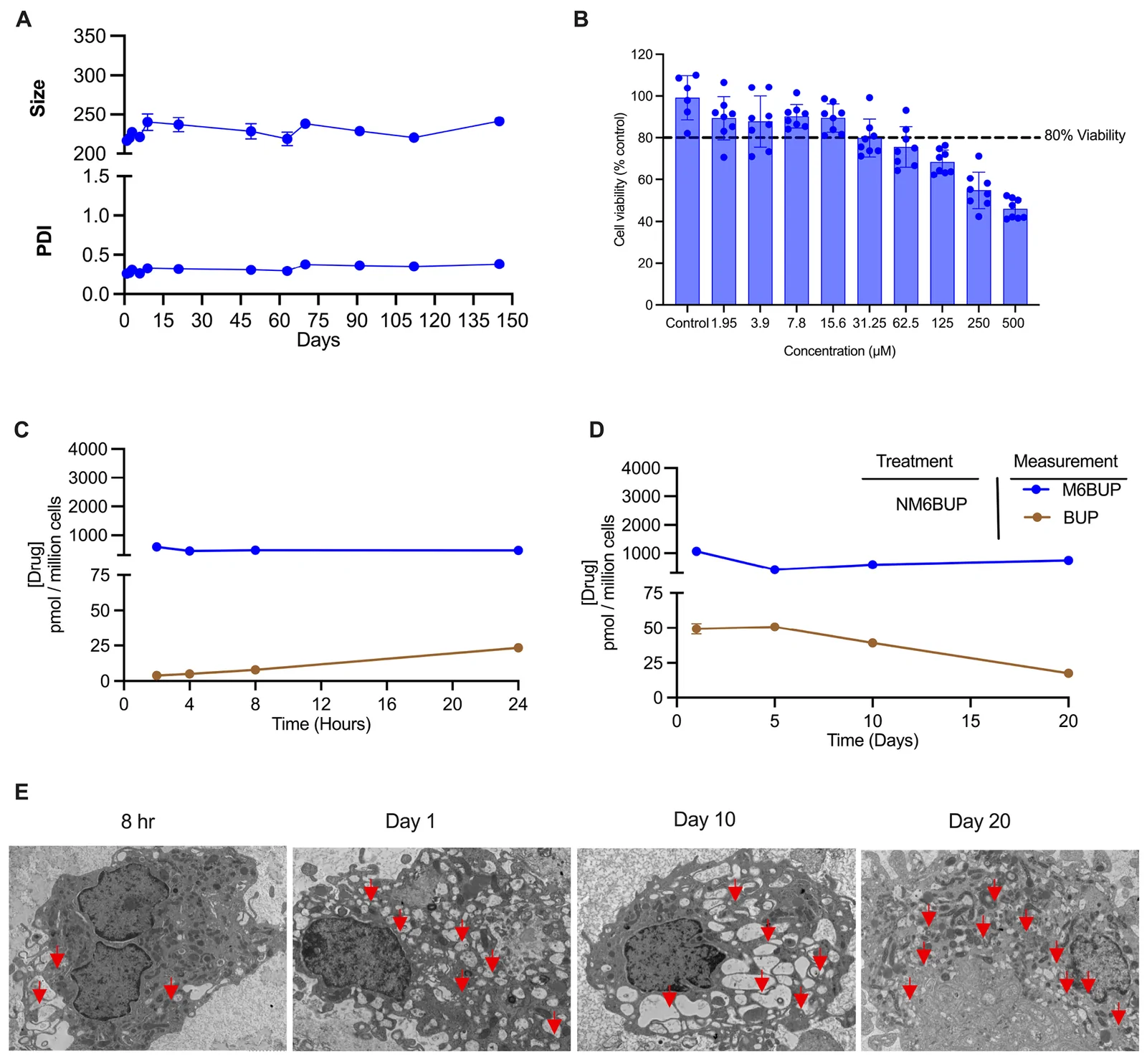
M6BUP free base aqueous nanosuspension production and characterization. **A** Stability of M6BUP nanosuspension (NM6BUP) at room temperature was evaluated using dynamic light scattering (DLS) over an observation period of 145 days. Data represent mean ± SEM (n = 3 independent replicates). **B** Viability of human monocyte-derived macrophages (MDMs) following 24 h exposure to increasing concentrations of NM6BUP (1.95–500 μM), normalized to untreated controls. Data are shown as mean ± SD (n = 4 biological replicates). **C** Prodrug uptake and activation by MDMs following 24 h exposure to 25 μM of NM6BUP. **D** Intracellular retention of the prodrug and BUP metabolite in MDMs over 20 days following an 8 h exposure to 25 μM NM6BUP. Intracellular drug levels were quantified by UPLC-UV/Vis. **C D** Data are expressed as mean ± SEM (n = 3 biological replicates). **E** Transmission electron microscopy (TEM) images of MDMs collected at 8 h, 1-, 10-, and 20-days post-treatment with NM6BUP for 8-hrs, demonstrating intracellular localization of the drug particles. **A–D** were independently repeated twice with consistent results. Source data are provided in the Figshare database under Digital Object Identifier (DOI) code (https://doi.org/10.6084/m9.figshare.34052919 )

**Figure 3:**
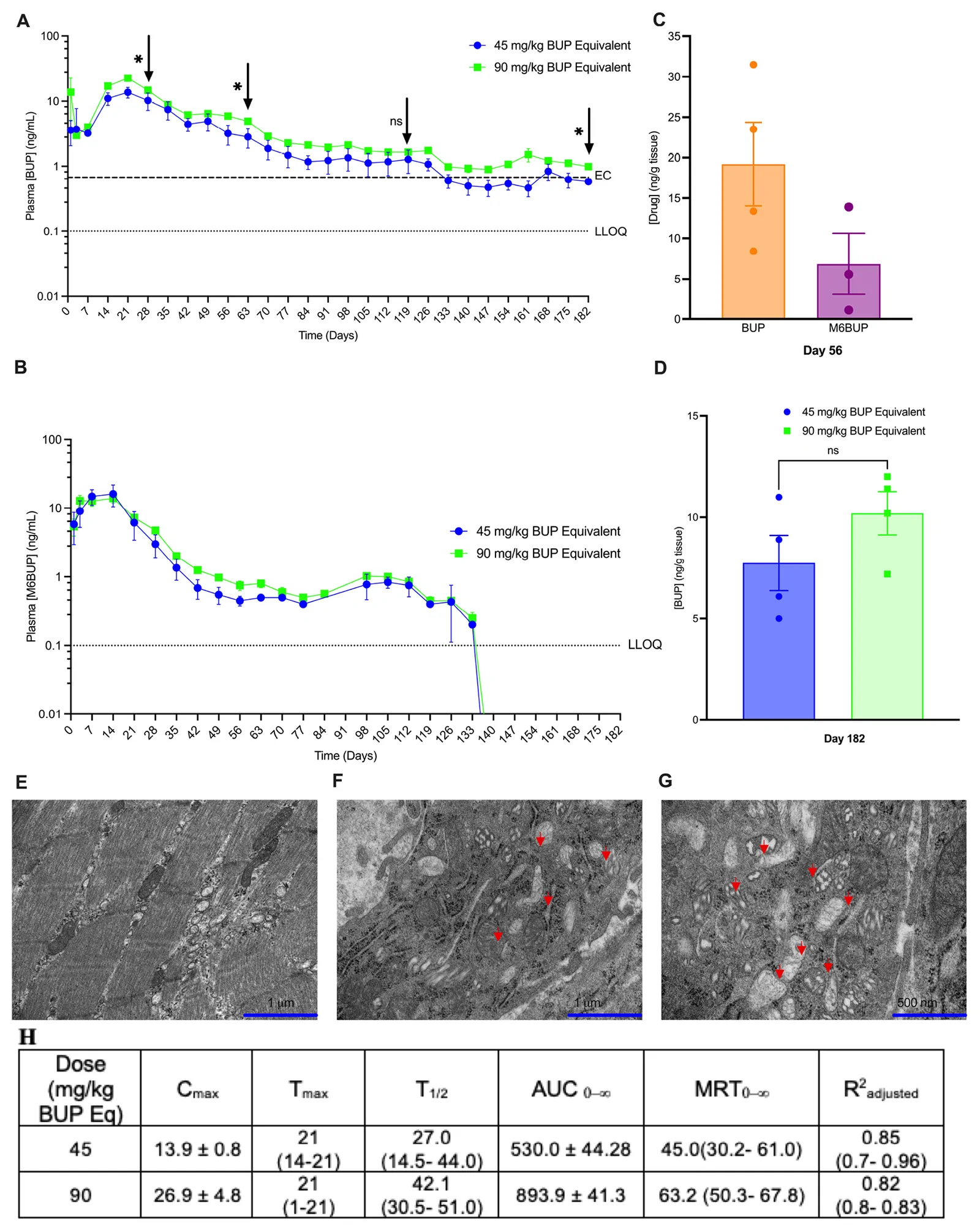
PK profiles for NM6BUP in Sprague Dawley rats. **A** Plasma concentration of BUP and **B** M6BUP following a single IM injection of NM6BUP at 45 or 90 mg/kg BUP-equivalent dose into the caudal thigh muscle of Sprague-Dawley rats. The study was initiated with n = 8 animals (45 mg/kg) and n = 4 animals (90 mg/kg). On day 56, 4 animals in the 45 mg/kg BUP equivalent group were sacrificed for brain drug level quantitation. All the other animals were sacrificed on day 182. BUP levels on Day 11, 28, 56, 119, and 182 were compared between treatment groups using one way ANOVA followed by Turkey’s multiple comparison. **C** Brain BUP and M6BUP concentrations on day 56 following IM administration of 45 mg/kg BUP-equivalent NM6BUP (n = 4 animals). **D** Brain BUP concentrations on day 182 following IM administration of 45 or 90 mg/kg BUP-equivalent NM6BUP (n = 4 animals/group). Statistical comparisons were performed using unpaired two-tailed Student’s t-test with Welch’s correction. **E–G** TEM images of injection-site skeletal muscle collected on day 182 from control animals and NM6BUP-treated rats (90 mg/kg BUP-equivalent). **E** Control and **F** 90 mg/kg BUP equivalent dose. **G** higher magnification of **F**. Higher-magnification images reveals cellular infiltration and endosomal sequestration of nanosuspension material (red arrows). Scale bars: 1 μm **E–F**, 500 nm **G**. Representative images are shown (n =4 biologically independent animals/group). **H** Non-compartmental pharmacokinetic parameters derived from plasma BUP concentrations after a single IM injection of NM6BUP at 45 or 90 mg/kg BUP-equivalent dose. C_max_, peak plasma concentration; T_max_, time to C_max_; T_½_, elimination half-life; AUC_0–∞_, area under the concentration–time curve; MRT_0–∞_, mean residence time. Data are expressed as mean ± SEM for C_max_ and AUC_0–∞_ and, as median (minimum–maximum) for for T_max_, T_½_, MRT_0–∞_, and R^2^_adjusted_. Source data are provided in the Figshare database under Digital Object Identifier (DOI) code ( https://doi.org/10.6084/m9.figshare.34052967 )

**Figure 4:**
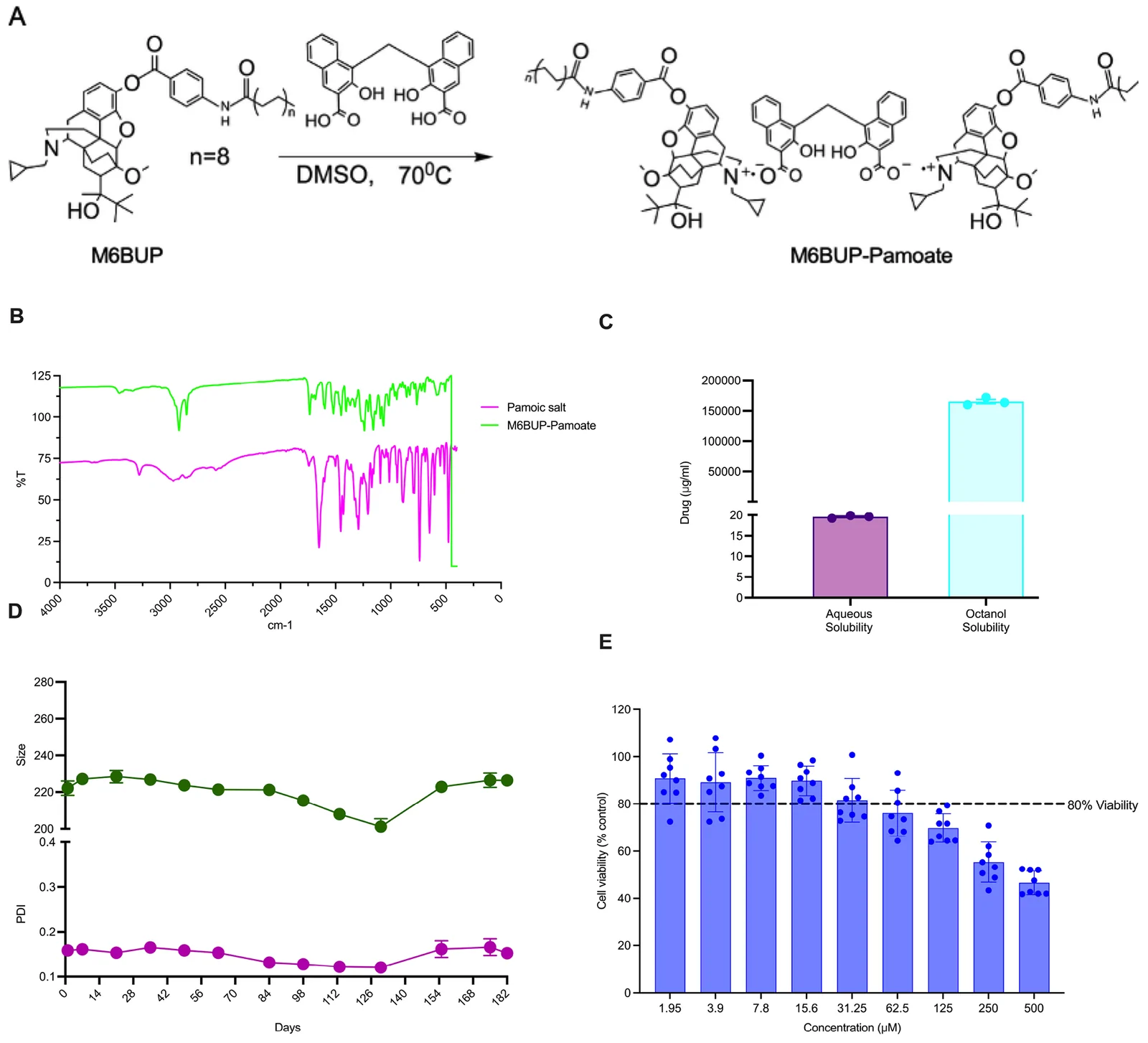
Synthesis and characterization of M6BUP-Pamoate. **A** Formation of M6BUP-Pamoate via hydrophobic ion-pairing of M6BUP with pamoic acid in DMSO at 70°C followed by aqueous precipitation. **B** Overlay of FT-IR spectra of M6BUP-Pamoate and pamoic acid, confirming characteristic functional groups associated with ion-pair formation. **C** Aqueous and octanol solubility of M6BUP-Pamoate quantified by LC-MS/MS. Data are presented as mean ± SEM (n = 3 independent replicates). **D** Long-term particle size and polydispersity index (PDI) stability of NM6BUP-Pamoate nanosuspension monitored by dynamic light scattering (DLS) over 182 days at room temperature (mean ± SEM, n = 3 independent replicates). **E** MDM viability following 24 h exposure to increasing concentrations of NM6BUP-Pamoate (1.95–500 μM), expressed as mean ± SEM (n = 4 biological replicates). Source data are provided in the Figshare database under Digital Object Identifier (DOI) code (https://doi.org/10.6084/m9.figshare.34052976 )

**Figure 5:**
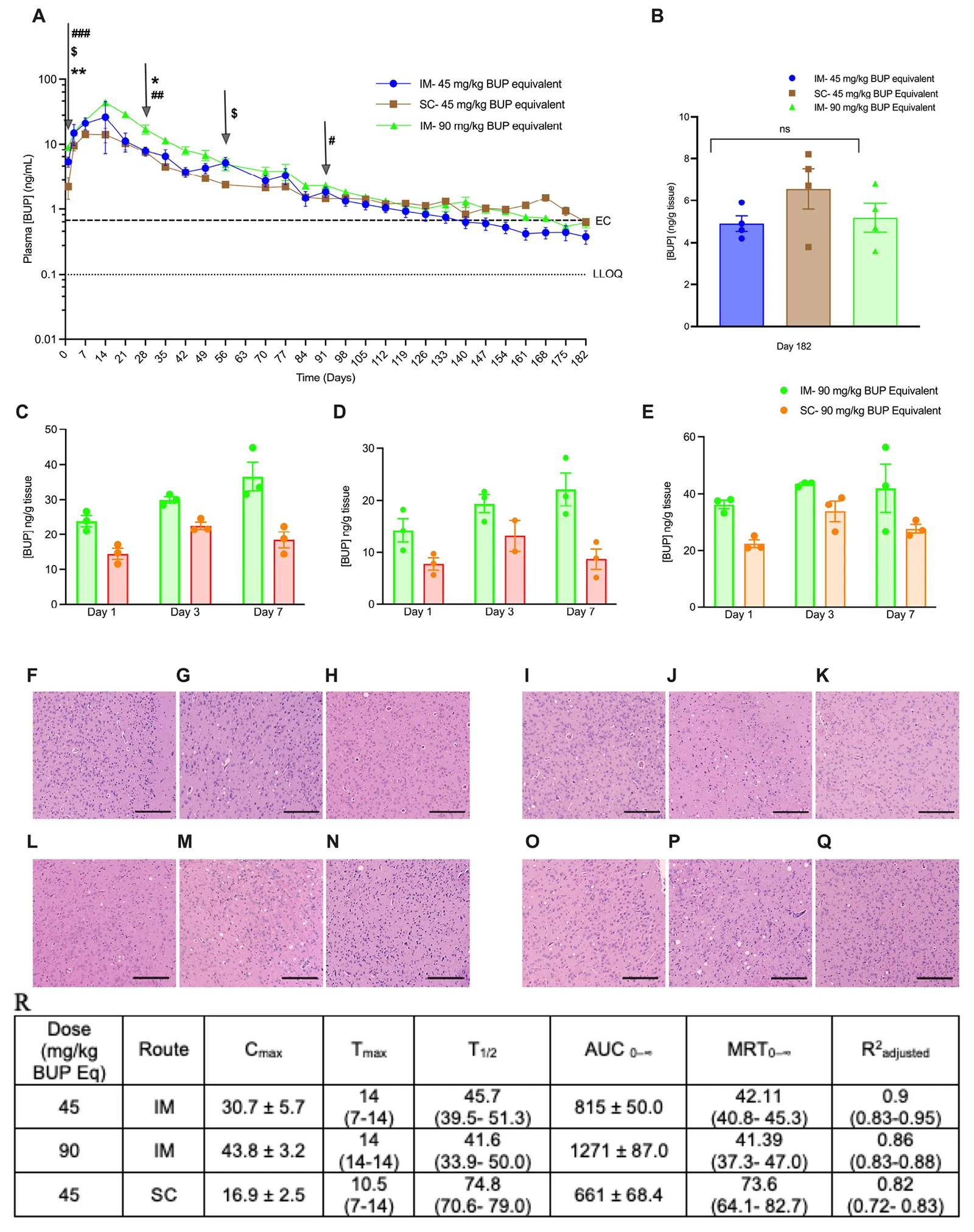
PK profiles for NM6BUP-Pamoate in Sprague-Dawley rats. **A** Plasma BUP concentration–time profiles and **B** brain BUP concentrations on day 182 following a single intramuscular (45 or 90 mg/kg BUP-equivalent) or subcutaneous (45 mg/kg BUP-equivalent) injection of NM6BUP-Pamoate in Sprague-Dawley rats (n = 4 animals/group). BUP levels on Day 1, 28, 56, 91, 119, and 182 were compared between treatment groups using one way ANOVA followed by Turkey’s multiple comparison. **C-E** Regional brain distribution of BUP (**C** cortex, **D** cerebellum, **E** midbrain) on days 1, 3, and 7 following a single IM or SC injection at 90 mg/kg BUP-equivalent dose (n = 4 animals/group). **F–Q** Representative hematoxylin and eosin (H&E)-stained sections of the cortex and midbrain at ×20 magnification on days 1 and 14. **F–H** Cortex on day 1: **F** vehicle control, **G** no-injection control, and **H** IM injection of NM6BUP-pamoate. **I–K** Cortex on day 14: **I** vehicle control, **J** no-injection control, and **K** IM injection of NM6BUP-pamoate. **L–N** Midbrain on day 1: **L** vehicle control, **M** no-injection control, and **N** IM injection of NM6BUP-pamoate. **O–Q** Midbrain on day 14: **O** vehicle control, **P** no-injection control, and **Q** IM injection of NM6BUP-pamoate. **R** Non-compartmental pharmacokinetic parameters following IM or SC NM6BUP-pamoate administration. C_max_, peak plasma concentration; T_max_, time required for C_max_, T_½_, elimination half-life; AUC_0–∞_, area under the plasma concentration time curve (AUC) from 0 hours to infinity; MRT_0–∞_, mean residence time from 0 hours to infinity. Results are expressed as mean ± SEM except T_1/2_ T_max_ , MRT_0–∞_ , R^2^_adjusted_ is expressed as median days (minimum-maximum). **A-E** Results are expressed as mean ± SEM. Source data are provided in the Figshare database under Digital Object Identifier (DOI) code ( https://doi.org/10.6084/m9.figshare.34052979 )

## Data Availability

Source data are provided in the Figshare database under Digital Object Identifier.

## References

[1] Volkow ND, Blanco C (2021) The changing opioid crisis: development, challenges and opportunities. Mol Psychiatry 26(1):218–233 10.1038/s41380-020-0661-4 PMC7398847 32020048

[2] Merianne R, Spencer MPH, Matthew F, Garnett MPH, Arialdi M, Miniño MPH (2024) Drug Overdose Deaths in the United States, 2002–2022

[3] Stein MD (2022) Retention in care for persons with opioid use disorder transitioning from sublingual to injectable buprenorphine. J Subst Abuse Treat 136:108661 10.1016/j.jsat.2021.108661 34801283

[4] Shulman M, Wai JM, Nunes EV (2019) Buprenorphine Treatment for Opioid Use Disorder: An Overview. CNS Drugs 33(6):567–580 10.1007/s40263-019-00637-z PMC6585403 31062259

[5] Gottlieb DJ (2023) A comparison of mortality rates for buprenorphine versus methadone treatments for opioid use disorder. Acta Psychiatr Scand 147(1):6–15 10.1111/acps.13477 35837885

[6] Kimber J (2015) Mortality risk of opioid substitution therapy with methadone versus buprenorphine: a retrospective cohort study. Lancet Psychiatry 2(10):901–908 10.1016/S2215-0366(15)00366-1 26384619

[7] Morgan JR (2019) Overdose following initiation of naltrexone and buprenorphine medication treatment for opioid use disorder in a United States commercially insured cohort. Drug Alcohol Depend 200:34–39 10.1016/j.drugalcdep.2019.02.031 PMC6613830 31082666

[8] Timko C (2016) Retention in medication-assisted treatment for opiate dependence: A systematic review. J Addict Dis 35(1):22–35 10.1080/10550887.2016.1100960 PMC6542472 26467975

[9] Lee KW (2025) Initiation and Dosing of Extended-Release Buprenorphine: A Narrative Review of Emerging Approaches for Patients Who Use Fentanyl. Subst Abuse Rehabil 16:71–82 10.2147/SAR.S516138 PMC11954470 40162321

[10] Lintzeris N, Dunlop AJ (2025) Buvidal/Brixadi - a long-acting injectable buprenorphine formulation for the treatment of opioid dependence. Pain Manag 15(12):897–910 10.1080/17581869.2025.2555164 PMC12674366 40891214

[11] Nunes EV (2024) Extended-Release Injection vs Sublingual Buprenorphine for Opioid Use Disorder With Fentanyl Use: A Post Hoc Analysis of a Randomized Clinical Trial. JAMA Netw Open 7(6):e2417377 10.1001/jamanetworkopen.2024.17377 PMC11200143 38916892

[12] Peckham AM (2021) Real-world outcomes with extended-release buprenorphine (XR-BUP) in a low threshold bridge clinic: A retrospective case series. J Subst Abuse Treat 126:108316 10.1016/j.jsat.2021.108316 34116808

[13] Andrilla CHA (2019) Geographic Distribution of Providers With a DEA Waiver to Prescribe Buprenorphine for the Treatment of Opioid Use Disorder: A 5-Year Update. J Rural Health 35(1):108–112 10.1111/jrh.12307 29923637

[14] Reddy IA (2024) Provider Perceptions Toward Extended-Release Buprenorphine for Treatment of Opioid Use Disorder. J Addict Med 18(5):540–545 10.1097/ADM.0000000000001320 PMC11446660 38829032

[15] Otte A (2025) Long-Acting injectable buprenorphine PLGA microparticle formulation. Int J Pharm 683:126006 10.1016/j.ijpharm.2025.126006 PMC12406168 40730319

[16] Smith L (2017) Probuphine (Buprenorphine) Subdermal Implants for the Treatment Of Opioid-Dependent Patients. P t 42(8):505–508 PMC5521298 28781503

[17] Barnwal P (2017) Probuphine^®^ (buprenorphine implant): a promising candidate in opioid dependence. Ther Adv Psychopharmacol 7(3):119–134 10.1177/2045125316681984 PMC5354129 28348732

[18] Aravindan L (2025) Pharmacogenomics of buprenorphine - a narrative review. Pharmacogenomics 26(7–9):263–270 10.1080/14622416.2025.2546770 PMC12427432 40908813

[19] Wang J-J (2009) Lipid nanoparticles with different oil/fatty ester ratios as carriers of buprenorphine and its prodrugs for injection. Eur J Pharm Sci 38(2):138–146 10.1016/j.ejps.2009.06.008 19591929

[20] Quach T (2022) Triglyceride-Mimetic Prodrugs of Buprenorphine Enhance Oral Bioavailability via Promotion of Lymphatic Transport. Front Pharmacol 13:879660 10.3389/fphar.2022.879660 PMC9039622 35496278

[21] Chin LS (2005) An esterification method for synthesizing prodrugs of buprenorphine. Acta Anaesthesiol Taiwan 43(4):223–229 16450597

[22] Ullah Nayan M (2023) Advances in long-acting slow effective release antiretroviral therapies for treatment and prevention of HIV infection. Adv Drug Deliv Rev 200:115009 10.1016/j.addr.2023.115009 37451501

[23] Edwards GG (2023) Long-Acting Injectable Therapy for People with HIV: Looking Ahead with Lessons from Psychiatry and Addiction Medicine. AIDS Behav 27(1):10–24 10.1007/s10461-022-03817-z PMC9443641 36063243

[24] Bardi F (2025) Clinical Outcomes in Patients with Schizophrenia Treated with Long-Acting Injectable vs. Oral Antipsychotics: A Naturalistic Study. Healthc (Basel), 13(14) 10.3390/healthcare13141709 PMC12295619 40724735

[25] Chien ST, Suydam IT, Woodrow KA (2023) Prodrug approaches for the development of a long-acting drug delivery systems. Adv Drug Deliv Rev 198:114860 10.1016/j.addr.2023.114860 PMC10498988 37160248

[26] Deodhar S (2022) Transformation of dolutegravir into an ultra-long-acting parenteral prodrug formulation. Nat Commun 13(1):3226 10.1038/s41467-022-30902-7 PMC9184486 35680875

[27] Kulkarni TA (2020) A year-long extended release nanoformulated cabotegravir prodrug. Nat Mater 19(8):910–920 10.1038/s41563-020-0674-z PMC7384935 32341511

[28] Nayan MU (2026) An ultra-long-acting dimeric bictegravir prodrug defined by a short pharmacokinetic tail. Nat Commun10.1038/s41467-026-68501-5 PMC12916993 41565650

[29] Cobb DA (2021) Transformation of tenofovir into stable ProTide nanocrystals with long-acting pharmacokinetic profiles. Nat Commun 12(1):5458 10.1038/s41467-021-25690-5 PMC8445934 34531390

[30] Mitchell M (2013) Single- and Multiple-Dose Pharmacokinetic, Safety, and Tolerability Profiles of Olanzapine Long-Acting Injection: An Open-Label, Multicenter, Nonrandomized Study in Patients With Schizophrenia. Clin Ther 35(12):1890–1908 10.1016/j.clinthera.2013.09.023 24184052

[31] Dowell D (2022) CDC Clinical Practice Guideline for Prescribing Opioids for Pain - United States, 2022. MMWR Recomm Rep 71(3):1–95 10.15585/mmwr.rr7103a1 PMC9639433 36327391

[32] Ballantyne JC, Mao J (2003) Opioid therapy for chronic pain. N Engl J Med 349(20):1943–1953 10.1056/NEJMra025411 14614170

[33] Spencer MR, Miniño GM (2024) AM. Drug overdose deaths in the United States, 2002–2022. NCHS Data Brief, no 491. Hyattsville, MD: National Center for Health Statistics. 10.15620/cdc:135849

[34] Meyers K (2014) High interest in a long-acting injectable formulation of pre-exposure prophylaxis for HIV in young men who have sex with men in NYC: a P18 cohort substudy. PLoS ONE 9(12):e114700 10.1371/journal.pone.0114700 PMC4263701 25502768

[35] Cobb DA (2020) Long-acting approaches for delivery of antiretroviral drugs for prevention and treatment of HIV: a review of recent research. Expert Opin Drug Deliv 17(9):1227–1238 10.1080/17425247.2020.1783233 PMC7442675 32552187

[36] Masson CL (2025) Barriers to Buprenorphine Treatment Among People Experiencing Homelessness: A Qualitative Study from the Provider Perspective. J Urban Health 102(2):465–475 10.1007/s11524-025-00967-y PMC12031702 40148731

[37] Weesner E (2022) Injection Site Reaction to Extended-Release Buprenorphine (Sublocade(^®^)) for Opioid Use Disorder Fourteen Days after Administration. Kans J Med 15(2):302–304 10.17161/kjm.vol15.17931 PMC9409986 36042837

[38] Reddy IA (2024) Provider perceptions toward extended-release buprenorphine for treatment of opioid use disorder. J Addict Med 18(5):540–545 10.1097/ADM.0000000000001320 PMC11446660 38829032

[39] Darville N (2014) Intramuscular administration of paliperidone palmitate extended-release injectable microsuspension induces a subclinical inflammatory reaction modulating the pharmacokinetics in rats. J Pharm Sci 103(7):2072–2087 10.1002/jps.24014 24845884

[40] Heise T (2014) Impact of injection speed and volume on perceived pain during subcutaneous injections into the abdomen and thigh: a single-centre, randomized controlled trial. Diabetes Obes Metab 16(10):971–976 10.1111/dom.12304 24720741

[41] Trezza C (2015) Formulation and pharmacology of long-acting cabotegravir. Curr Opin HIV AIDS 10(4):239–245 10.1097/COH.0000000000000168 PMC5638427 26049948

[42] Darville N (2016) The effect of macrophage and angiogenesis inhibition on the drug release and absorption from an intramuscular sustained-release paliperidone palmitate suspension. J Control Release 230:95–108 10.1016/j.jconrel.2016.03.041 27067365

